# When does it pay off to prime for defense? A modeling analysis

**DOI:** 10.1111/nph.14771

**Published:** 2017-09-11

**Authors:** Jacob C. Douma, Peter J. Vermeulen, Erik H. Poelman, Marcel Dicke, Niels P. R. Anten

**Affiliations:** ^1^ Centre for Crop Systems Analysis Wageningen University Droevendaalsesteeg 1 6708PB Wageningen the Netherlands; ^2^ Laboratory of Entomology Wageningen University Droevendaalsesteeg 1 6708PB Wageningen the Netherlands

**Keywords:** community, fitness, insect herbivory, plant competition, priming, volatiles

## Abstract

- Plants can prepare for future herbivore attack through a process called priming. Primed plants respond more strongly and/or faster to insect attack succeeding the priming event than nonprimed plants, while the energetic costs of priming are relatively low.
- To better understand the evolution of priming, we developed a simulation model, partly parameterized for *Brassica nigra* plants, to explore how the fitness benefits of priming change when plants are grown in different biotic environments.
- Model simulations showed that herbivore dynamics (arrival probability, arrival time, and feeding rate) affect the optimal duration, the optimal investment and the fitness benefits of priming. Competition for light increases the indirect costs of priming, but may also result in a larger payoff when the nonprimed plant experiences substantial leaf losses.
- This modeling approach identified some important knowledge gaps: herbivore arrival rates on individual plants are rarely reported but they shape the optimal duration of priming, and it would pay off if the likelihood, severity and timing of the attack could be discerned from the priming cue, but it is unknown if plants can do so. In addition, the model generated some testable predictions, for example that the sensitivity to the priming cue decreases with plant age.

## Introduction

Plants can prepare for future herbivore attack through a process called priming (Frost *et al*., 2007; Heil & Silva Bueno, 2007; Rodriguez‐Saona *et al*., 2009; Kim & Felton, 2013). Priming is defined as the process in which a temporally limited stimulus prepares and modifies the response to an upcoming stress event (Conrath *et al*., 2006, 2015; Frost *et al*., 2008; Hilker *et al*., 2015; see Table 1 for a glossary). For example, it has been observed that plants previously exposed to volatiles from insect‐infested neighbors were subsequently more resistant to insect herbivores (Kessler *et al*., 2006; Peng *et al*., 2011; Karban *et al*., 2013b).

**Table 1:** Glossary of terms used in this paper

Term	Explanation
Priming	The process in which a temporally limited stimulus prepares and modifies the response to an upcoming stress event (Conrath *et al*., 2006; Frost *et al*., 2008; Hilker *et al*., 2015)
Priming cue	A temporally limited stimulus that leads to priming
Priming strategy	The strategy of responding to a priming cue and becoming primed
Nonpriming strategy	The strategy of not responding to priming cue and hence not becoming primed (thus staying naïve)
Trigger event	The upcoming stress event for which the priming prepares
Direct costs	The energetic investment in priming
Indirect costs	The loss in net photosynthesis of a primed plant compared with a nonpriming plant after correcting for the direct costs of priming

Primed plants have been shown to exhibit faster and/or stronger responses to herbivore attack compared with plants that do not prime, and priming can lead to decreased caterpillar growth rates (Peng *et al*., 2011), increased pathogen resistance (van Hulten *et al*., 2006; Walters *et al*., 2008) and possibly increased movement of insect herbivores to more palatable plants (Morrell & Kessler, 2016). Therefore, priming is hypothesized to be beneficial for a plant as it may lead to a faster response to the upcoming attack (hereafter called the trigger event), while its costs are estimated to be relatively low compared with immediate defense (Bruce *et al*., 2007; Frost *et al*., 2008). For example, van Hulten *et al*. (2006) showed that the relative growth rate (RGR) of primed plants decreased by up to 27% compared with control plants, while RGR was much more reduced when defense was activated immediately (39–44%). Priming may, therefore, help the plant to prepare for future events while economizing on resources (van Hulten *et al*., 2006; Frost *et al*., 2007, 2008; van Hulten *et al*., 2006).

Understanding the metabolic mechanisms underlying priming has been a major effort, mainly in plant–pathogen systems (Conrath *et al*., 2015; Hilker *et al*., 2015; but see Kim & Felton, 2013 for an overview of plant–herbivore systems). Those studies showed that priming may take place at the epigenetic level, the transcriptome level, the posttranscription level and the physiological level. Also, it has been shown that priming may lead to a stronger (Jakab *et al*., 2005; Ton *et al*., 2007), faster (Jakab *et al*., 2005) or earlier (Ton *et al*., 2007) response to the trigger event. It is very likely that the type of response pattern, as well as the level at which priming occurs, affects the costs of priming, but to date it is unknown whether this is the case.

To fully understand the functional significance and evolution of priming, the aforementioned costs and benefits should be evaluated in the ecological context in which priming occurs. Yet, this is poorly understood (Martinez‐Medina *et al*., 2016). One important factor that has been identified is the severity of the attack after the priming cue. For example, Walters *et al*. (2008) showed that priming was only beneficial when pathogen pressure on barley (*Hordeum vulgare*) was high. A number of other important factors can be identified. First, competition for resources may amplify the costs of priming (Rillig *et al*., 2015). A small investment in priming early in the growing season may lead to a reduced investment in resource acquisition components (leaves and roots) which gives a competitive disadvantage that accumulates over time. Thus, we expect that the priming costs that were estimated by van Hulten *et al*. (2006) will be an underestimation for plants growing in competition. Second, herbivore movement dynamics may determine the time over which the plant should stay primed and the period over which priming costs are paid. Third, the benefit of priming will be dependent on the degree to which the primed plant responds faster to the trigger event compared with a nonpriming strategy. Finally, theory on plant plasticity predicts that the reliability of the priming cue will affect the adaptive benefit of priming (DeWitt *et al*., 1998; Karban *et al*., 1999; Schmitt *et al*., 2003). More precisely, error management theory predicts that cue reliability in addition to the costs of a wrong response determines whether it is beneficial to respond to the cue (Frost *et al*., 2008; Orrock *et al*., 2015).

In summary, some of the components that (are hypothesized to) affect the benefits of priming have been studied in isolation, but a coherent and quantitative framework in which the relationships that govern the costs and benefits of priming can be studied is lacking. Because the number of possible interactions (and number of experiments) is manifold, a modeling analysis can help to identify knowledge gaps, find ecological conditions in which priming is beneficial and provide directions for future experimental research.

The aim of this study was to elucidate the effects of physiological and environmental factors, and their interplay, on the benefits of priming. We did this by modeling the quantitative relationships and processes that govern the costs and fitness benefits of priming. In particular, we explored how the optimal level of priming (i.e. the level of priming associated with maximum plant fitness) varies with the degree of plant competition, herbivore feeding rate, the time between the priming and the trigger event, the time for which the plant stays primed, plant age and the response pattern of the primed plant.

## Description

### Model description

**Figure 1:**
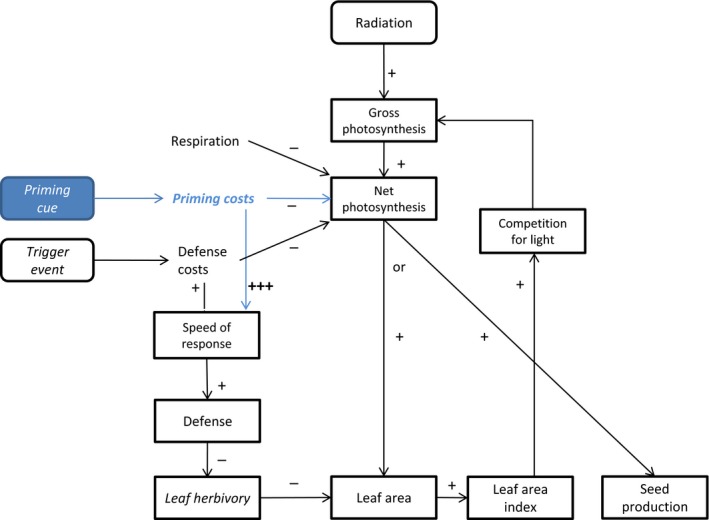
Schematic representation of the model. Two strategies are compared: a priming strategy (blue) that responds to a priming cue and a nonpriming strategy that does not respond to a priming cue. When the priming cue occurs, the priming strategy incurs priming costs. When the trigger event occurs, both strategies pay defense costs. However, the build‐up of defense is stronger in the priming strategy compared with the nonpriming strategy, initially leading to reduced leaf consumption. Thus, herbivory affects the loss of leaf area, leading to a reduction in light interception. The + and − indicate the influence of one process on another. Parameters/variables for which the impact on the benefits of priming will be explored are indicated in italics.

**Figure 2:**
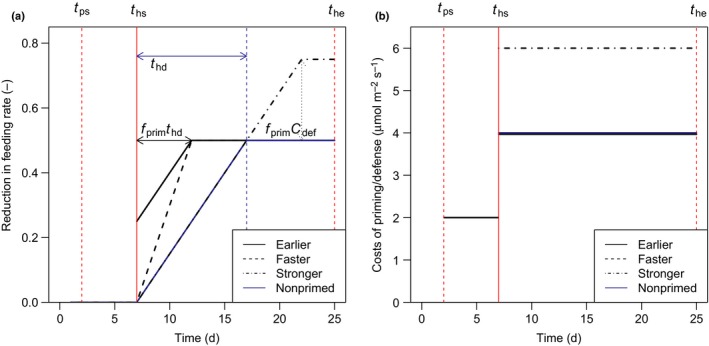
Following Hilker *et al*. (2015), the primed state may lead to (1) an earlier build‐up of defensive compounds, (2) a faster build‐up of defensive compounds or (3) a stronger build‐up of defensive compounds compared with a naïve plant that experiences a delay (*t*_hd_) before defenses are at the desired level (a). The degree to which the primed strategy responds faster, more strongly or earlier is related to the costs of priming (*f*_prim_). The priming costs for the three types of response are equal during the priming phase and are paid from when the priming cue occurs (*t*_ps_) until the trigger event occurs (*t*_hs_) (b). The defense costs are equal for the nonprimed and the ‘earlier’ and ‘faster’ strategies and are paid from the start of the trigger event (*t*_ps_) until the end (*t*_pe_) (the faster strategy is not visible as it overlies the earlier and nonprimed lines). The primed plant that responds ‘more strongly’ to the trigger event compared with a nonprimed plant experiences higher defense costs.

A model is used to compute plant fitness for two different strategies of a focal plant: a plant either responds to the priming cue by entering the primed state or does not respond and remains naïve (Fig. 1). The primed state leads to a different defense response to the trigger event compared with a naïve plant. The way in which this response differs depends on the scenario explored (see Fig. 2). Inputs of the model that are varied are plant density, herbivore feeding rate, energetic costs of priming, the timing of the priming cue relative to the timing of the trigger event, and the type of priming response to the trigger event. A plant competes with other plants if the number of plants per m^2^ is larger than 1, that is, *D* > 1. We assume that the plants with which the focal plant competes do not respond to the priming cue and stay in the naïve state after the priming event (nonpriming strategy).

The core of the model is based on Vermeulen (2015). Plant fitness (*F*_i_) is defined as the investment of assimilates in reproduction over the lifespan of the plant (*t*_s_) under the assumption that the investment in reproduction relates linearly to the number of offspring ((Eqn 1)). It is further assumed that it is a determinate species and that it completely switches from the vegetative to the generative phase at a predefined point in time (*t*_f_; thus, after *t*_f_ assimilates are invested in reproduction). For parameters and their values, see Table 2. Model assumptions are listed in Supporting Information Table S1. Plant fitness can then be found as:

**Table 2:** Overview of the parameters and variables used in the model, and their values and sources

Parameter	Description	Units	Value	Source
*t*_f_	Flowering onset	d	80^a^	Vermeulen (2015)
*t*_s_	End of the growing season	d	180	Assumed
*t*_hs_	Onset of herbivory	d	3–30	Varied in model
*t*_he_	End of herbivory		180	E. H. Poelman (unpublished data)
*t*_hd_	Delay in defense build‐up	d	6 d	León *et al*. (2001); Heil & Ton (2008); Dicke *et al*. (2009); Stam *et al*. (2014)
*t*_ps_	Onset of priming	d	*t*_ps_ < *t*_hs_	Varied in model
*t*_pe_	End of priming phase	d		Varied in model
*t*_pd_	Duration of priming event	d		Varied in model
*L*_ue_	Light use efficiency	μmol C μmol^−1^ intercepted photons	0.020	Vermeulen (2015)
*k*	Light extinction coefficient	−	0.8	Vermeulen (2015)
*I*_0_	Photon flux density above the canopy	μmol photons m^−2^ surface area s^−1^	1000	Vermeulen (2015)
α	Competition coefficient	−	1–1.5	Varied in model
*D*	Plant density	No. m^−2^	1–8	Varied in model
LAI_t0_	Seedling leaf area	m^2^ m^−2^	0.01	Vermeulen (2015)
*F*_LA_	Fraction of mass in leaves	0.33		
*C*_LAI_	Costs of producing new leaf area	μmol C m^−2^ leaf area	137 714	Vermeulen (2015)
*C*_l_	Maintenance costs of a unit leaf area	μmol C m^−2^ s^−1^	3.32	Vermeulen (2015)
*F*_pot_	Feeding rate (in absence of induced defense)	m^2^ s^−1^	1e‐08–3.5e‐07 m^2^ s^−1^	J. de Vries (unpublished data); E. H. Poelman (unpublished data)
*C*_def_	Costs of maintaining defense	μmol C m^−2^ s^−1^	−1 μmol C m^−2^ s^−1^	Assumed, varied in model
*f*_prim_	Costs of priming of the focal plant relative to *C*_def_	−	0–1	Varied in model

a Average of optimal flowering time given length of growing season.

If *t*_f_ ≤ *t* ≤ *t*_s_

(Eqn 1) $$F_{i}=\sum_{t_{f}}^{t_{s}}P_{\mathrm{net},i(t)}$$

(*i*, plant number; *t*, time.)

As long as *t* < *t*_f_, the leaf area is built up over time on one unit of ground surface (leaf area index (LAI); m^2^ leaf m^−2^ soil surface) (Eqn 2). The model calculates the leaf area production of a plant (*i*) that competes for light with a number of other individuals that occupy the same soil surface area. Thus, we assume homogeneous mixing of plants in the canopy.

(Eqn 2) $${\text{LAI}}_{i,(t+1)}={\text{LAI}}_{i,(t)}+\Delta {\text{LAI}}_{i,(t)}$$

The production of new leaf area at time *t* depends on the net photosynthesis at time *t* (*P*_net_; CO_2_ m^−2^ soil surface s^−1^), the costs of producing one unit of leaf area (*C*_LAI_; μmol C m^−2^ leaf), and the feeding rate of the herbivore ($Co_{r,i,(t)}$; m^2^ leaf s^−1^; (Eqn 3)). Stems and roots are not explicitly modeled, but the value for the costs of producing one unit of leaf area includes the costs for producing stems and roots needed to support the leaves (Vermeulen, 2015).

(Eqn 3) $$\Delta {\text{LAI}}_{i,(t)}=\left(\frac{P_{\mathrm{net},i(t)}}{C_{\mathrm{LAI}}}-Co_{r,i,(t)}{\text{LAI}}_{i,(t)}\right)\Delta t$$

Net photosynthesis is calculated both for the priming and the nonpriming strategies. For the nonpriming strategy, net photosynthesis is calculated from gross photosynthesis, leaf maintenance costs (*C*_l_; μmol C m^−2^ leaf s^−1^), and maintenance costs for defense against herbivores (*C*_def,i_; μmol C m^−2^ leaf s^−1^; (Eqn 2)). Maintenance costs for defense are only paid over the time for which the insect herbivores are present (between the onset of herbivory (*t*_hs_) and the end of herbivory (*t*_he_); referred to as the trigger event). Therefore, two phases can be distinguished: a phase where no herbivores are present (0 < *t* < *t*_hs_ or *t*_he_ < *t* ≤ *t*_s_) and a phase where herbivores are present (*t*_hs_ ≤ ≤ *t*_he_).

(Eqn 4) $$P_{\mathrm{net},i,(t)}=\left\{\begin{array}{cc} {\text{Fr}}_{i,(t)}I_{o}L_{\mathrm{ue}}\left(1-e^{-k\left(\sum_{i=1}^{D}{\text{LAI}}_{i,(t)}\right)}\right)-C_{l}{\text{LAI}}_{i(t)} & \text{if}\;0<t<t_{\mathrm{hs}}\;\text{or}\;t_{\mathrm{he}}<t\leq t_{s}\\ {\text{Fr}}_{i,(t)}L_{\mathrm{ue}}\left(1-e^{-k\left(\sum_{i=1}^{D}{\text{LAI}}_{i,(t)}\right)}\right)-C_{l}{\text{LAI}}_{i(t)}-C_{\mathrm{def},i}{\text{LAI}}_{i(t)} & \text{if}\;t_{\mathrm{hs}}\leq t\leq t_{\mathrm{he}} \end{array}\right.$$

For the priming strategy, a third phase is distinguished in addition to these two phases. After the onset of the priming cue (*t*_ps_), priming costs are being paid for *t*_pd_ days, until $t_{\mathrm{pe}}\left(t_{\mathrm{pe}}=t_{\mathrm{ps}}+t_{\mathrm{pd}}\right)$, but maximally until the start of the trigger event; if $t_{\mathrm{ps}}\leq t<\min\left(t_{\mathrm{ps}}+t_{\mathrm{pd}},t_{\mathrm{hs}}\right)$.When priming costs are not paid any more, the priming effect vanishes at the same speed as the priming was built up (Eqns (Eqn 10), (Eqn 11), (Eqn 12)). Priming costs (*f*_prim__,i_ [−]; (Eqn 5)) are expressed relative to defense costs because the defense and priming costs are uncertain from the literature, and in this way one can easily explore how the (optimal) benefits of priming relate to the benefits of inducible defense, either for a naïve plant that responds to the trigger event by inducing its defense (*f*_prim_ = 0) or for a plant that responds to the priming cue by inducing its defense (*f*_prim_ = 1).

(Eqn 5) $$P_{\mathrm{net},i,(t)}=\left\{\begin{array}{cc} {\text{Fr}}_{i,(t)}L_{\mathrm{ue}}\left(1-e^{-k\left(\sum_{i=1}^{D}{\text{LAI}}_{i,(t)}\right)}\right)-C_{l}{\text{LAI}}_{i(t)} & \text{if}\;0<t<t_{\mathrm{ps}}\;\text{or}\;\\ & \min\left(t_{\mathrm{pe}},t_{\mathrm{hs}}\right)<t<t_{\mathrm{hs}}\;\text{or}\;\\ & t_{\mathrm{he}}<t<t_{s}\\ {\text{Fr}}_{i,(t)}L_{\mathrm{ue}}\left(1-e^{-k\left(\sum_{i=1}^{D}{\text{LAI}}_{i,(t)}\right)}\right)-C_{l}{\text{LAI}}_{i(t)}-f_{\mathrm{prim},i}C_{\mathrm{def},i}{\text{LAI}}_{i(t)} & \text{if}\;t_{\mathrm{ps}}\leq t<\min\left(t_{\mathrm{pe}},t_{\mathrm{hs}}\right)\\ {\text{Fr}}_{i,(t)}L_{\mathrm{ue}}\left(1-e^{-k\left(\sum_{i=1}^{D}{\text{LAI}}_{i,(t)}\right)}\right)-C_{l}{\text{LAI}}_{i(t)}-C_{\mathrm{def},i}{\text{LAI}}_{i(t)} & \text{if}\;t_{\mathrm{hs}}\leq t<t_{\mathrm{he}} \end{array}\right.$$

Gross photosynthesis is calculated using the absorbed light and the light use efficiency, that is, photosynthesis per unit of absorbed light (*L*_ue_; μmol C μmol^−1^ intercepted photons). The absorbed light is described by a decreasing exponential function and is related to the amount of leaf area in the canopy (LAI; m^2^ leaf m^−2^ ground surface) and the light extinction coefficient (*k*, −): $1-e^{-k\left(\sum_{i=1}^{D}{\text{LAI}}_{i,(t)}\right)}$.

Fr represents the competition coefficient as it determines the share of plant *i* in generating canopy‐level gross photosynthesis (−). Fr is calculated from the ratio of the leaf area of plant *i* over the average leaf area in the canopy of *D* plants in 1 m^2^ and a competition coefficient *α* (Eqns 6, 7). Thus, the lower Fr, the lower the share of a plant's leaf area in the canopy. The competition coefficient α represents the extent to which competition is symmetric or asymmetric. If α equals 1 then competition is symmetric: proportionally higher LAI leads to proportionally higher gross photosynthesis. If α is larger than 1, plants with higher LAI relative to their competitors capture disproportionally more light.

(Eqn 6) $${\text{Fr}}_{i,(t)}=\frac{C\left({\text{LAI}}_{i,(t)}\right)}{C\left({\text{LAI}}_{i,(t)}\right)+\cdots +C\left({\text{LAI}}_{D,(t)}\right)}$$

(Eqn 7) $$C\left({\text{LAI}}_{i,(t)}\right)={\left(\frac{{\text{LAI}}_{i,(t)}}{\frac{\sum_{i=1}^{D}{\text{LAI}}_{i,(t)}}{D}}\right)}^{\alpha }$$

Leaf herbivory is introduced through the leaf feeding rate ($F_{i,(t)}$; m^2^ s^−1^). It is calculated separately for the primed and nonprimed plants. The feeding rate of the insect herbivores is only affected by the defense level of the plant. If the plant is not defended, the herbivores consume leaf area at their potential rate ($F_{\mathrm{pot},(t)}$; m^2^ leaf s^−1^). A nonprimed plant may respond to the trigger event by producing defensive compounds that reduce the feeding rate to $F_{i,(t)}$ (Eqn 8). The reduction in feeding rate is determined by the level of defense, $C_{\mathrm{def},i,(t)}$, and a parameter *b* (m^2^ leaf s μmol C^−1^) that scales the investment in defense to unit scale.

(Eqn 8) $$F_{i,(t)}=F_{\mathrm{pot},(t)}\left(1-b\;C_{\mathrm{def},i,(t)}\right)$$

Thus, we assume that the investment in defense is linearly related to the reduction in feeding rate: no investment results in the potential feeding rate, while a maximum investment leads to a 100% reduction of the feeding rate. The 100% reduction reflects the death of the herbivore and/or the movement of the herbivore to a more palatable stand. Furthermore, we assume that plants experience a delay (*t*_hd_; days) in the build‐up of defensive compounds. This delay represents the time needed to induce the biosynthetic pathway and get the defense level up to the desired level *C*_def,pot,i_ (Eqn 9). The time it takes to produce defensive compounds reported in the literature ranges from hours to days or even weeks (León *et al*., 2001; Heil & Ton, 2008; Dicke *et al*., 2009; Stam *et al*., 2014).

(Eqn 9) $$C_{\mathrm{def},i,(t)}=\min\left(C_{\mathrm{def},\mathrm{pot},i},\frac{C_{\mathrm{def},\mathrm{pot},i}}{t_{\mathrm{hd}}}\left(t-t_{\mathrm{hs}}\right)\right)$$

The priming strategy differs from the nonpriming strategy (also called naïve) in that the level of defensive compounds ($C_{\mathrm{def},i,(t)}$ and hence the reduction in feeding rate are larger and occur with less delay. Hilker *et al*. (2015) distinguished four types of priming response pattern: an earlier onset of the defense response after priming relative to a nonprimed plant (‘earlier’); a faster defense response upon the trigger event relative to a nonprimed plant (‘faster’); a stronger response to the trigger event relative to a nonprimed plant (‘stronger’); and a more sensitive response to the trigger event compared with a nonprimed plant. We only model the first three response types. The fourth response pattern is not modeled as we do not model the intensity of the priming cue. Thus, we ignore the possibility that the intensity of the cue may affect the level of priming (Niinemets *et al*., 2013). In the first two response patterns, the reduction in feeding rate is larger compared with a nonprimed plant because the desired defense level is reached earlier (Fig. 2; Eqns 11, 11). *t*_adv_ represents the number of days by which the primed plant reaches the desired level earlier compared with nonprimed plants.

To calculate *t*_adv_, we distinguish three possibilities in the priming phase: in the first scenario, the priming strategy is still building up its priming metabolism while the trigger event occurs. In this case, the advantage of the priming strategy is only *t*_hs_ − *t*_ps_ days. The second scenario occurs when the priming strategy has finished the build‐up of priming components and the trigger event occurs when the priming cue is still in the memory of the plant. Then *t*_adv_ is linearly related to the investment in priming: if *f*_prim,i_ = 1, the plant experiences no delay in building up its defensive compounds; if *f*_prim,i_ = 0, the plant experiences a delay similar to that experienced by nonprimed plants. The third scenario occurs when the priming strategy has finished its build‐up of priming components, the trigger event has not occurred yet, and the plant stops paying costs (when *t*_pe_ < *t*_hs_). In this case, we assume that *t*_adv_ decreases linearly with time at the same speed as it was built up (Eqn 12).

(Eqn 10) $$C_{\mathrm{def},i,(t)}=\min\left(C_{\mathrm{def},\mathrm{pot},i},\frac{C_{\mathrm{def},\mathrm{pot},i}}{t_{\mathrm{hd}}}\left(t+t_{\mathrm{adv}}-t_{\mathrm{hs}}\right)\right)$$

(Eqn 11) $$C_{\mathrm{def},i,(t)}=\min\left(C_{\mathrm{def},\mathrm{pot},i},\frac{C_{\mathrm{def},\mathrm{pot},i}}{t_{\mathrm{adv}}f_{\mathrm{prim},i}T_{\mathrm{hd}}}\left(t-t_{\mathrm{hs}}\right)\right)$$

with

(Eqn 12) $$\begin{array}{cc} t_{\mathrm{adv}}= & \min\left(\min\left(f_{\mathrm{prim},i}t_{\mathrm{hd}},t_{\mathrm{hs}}-t_{\mathrm{ps}}\right),\right.\\ & \max\left(0,\left(f_{\mathrm{prim},i}t_{\mathrm{hd}}-\left(t_{\mathrm{hs}}-t_{\mathrm{pe}}\right)\right)\right) \end{array}$$

The third response pattern reaches higher defense levels compared with the nonprimed plant. For all priming strategies, we assume that the priming costs (*f*_prim,i_ *C*_def,pot,i_) are equal (Eqn 5). In addition, the defense costs for the first two response patterns are exactly the same as for the nonprimed plants. By contrast, the defense costs of the third response pattern are *f*_prim,i_ *C*_def,pot,i_ higher.

(Eqn 13) $$C_{\mathrm{def},i,(t)}=\min\left(C_{\mathrm{def},\mathrm{pot},i}\left(1+f_{\mathrm{prim},i}\right),\frac{C_{\mathrm{def},\mathrm{pot},i}\left(1+f_{\mathrm{prim},i}\right)}{t_{\mathrm{hd}}\left(1+f_{\mathrm{prim},i}\right)}\left(t-t_{\mathrm{hs}}\right)\right)$$

### Parameterization

The theoretical model was parameterized for a fast‐growing annual and we assumed that light was the only limiting factor for growth. Growth parameters were taken from Vermeulen (2015): light use efficiency (*L*_ue_) was taken to be 0.02 μmol C μmol^−1^ intercepted photons, the cost of producing new leaf area (*C*_LAI_) was taken to be 137 714 μmol C m^−2^ leaf area, the light extinction coefficient (*k*) was 0.8 [−], and the maintenance cost of leaf area (*C*_l_) was 3.32 μmol C m^−2^ s^−1^. The *C*_LAI_ taken from Vermeulen (2015) was calculated on the basis of specific leaf area, the allocation of mass to leaves, the leaf carbon content and the construction costs of one gram of leaf. A growing season (*t*_s_) length of 180 d was chosen, corresponding to the growing season length for an annual plant, such as *Brassica nigra* L., in northern Europe. The shift from the vegetative to the flowering stage (*t*_f_) was absolute and set at 80 d – which is, in terms of fitness, the optimal flowering time for the specified growing season length and the other growth parameters (Vermeulen, 2015). Note that effect temperature on growth is not implemented in the model.

The average feeding rate of a single insect herbivore during its lifetime was set at 8 × 10^−9^ m^2^ leaf s^−1^ and was estimated from *Pieris rapae* L. caterpillars feeding on *B. nigra* (J. de Vries, unpublished data). Feeding rate was varied in the model. In the model, we do not distinguish different types of insect herbivores, as we assume that their differences will mainly be apparent in their differences in feeding rate. We assume that the number of leaf‐feeding herbivores during the growing season follows a lognormal pattern (i.e. initially the number of herbivores is low, reaching a maximum of on average 10.5 (3.5–17.6 95% observation confidence band) insects at 32 d, and then slowly declining (estimated from E. H. Poelman, unpublished data for *B. nigra*)). These data were also used to estimate the average time between the first arrival of an insect herbivore in a stand and subsequent herbivore attack on other plants in the stand. Its value was estimated to be 8.4 d (the observed minimum and maximum were 0 and 18, respectively). The plot average time between the first arrival and subsequent arrivals was maximally 15 d (E. H. Poelman, unpublished data for *B. nigra*). The delay in the build‐up of defenses of nonprimed plants, *t*_pd_, was set at 6 d (León *et al*., 2001; Heil & Ton, 2008; Dicke *et al*., 2009; Stam *et al*., 2014). Costs of defense, *C*_def_, were varied between 0 and 1 μmol C m^−2^ s^−1^. The costs of priming (*f*_prim_) were varied relative to the costs of defense and varied between 0 and 1. The costs of priming have been shown to be smaller than those of induced defense. van Hulten *et al*. (2006) showed that RGR decreased by up to 27% when plants were primed compared with nonprimed plants, depending on the age of the plant and the β‐aminobutyric acid dose applied, while Walters *et al*. (2008) could not detect a significant reduction in RGR when primed. Priming can occur through various mechanisms, some of which last longer (months) and increase in intensity over time, while others have a shorter lifetime and decrease in intensity with time (days; Karban & Shiojiri, 2009; Ding *et al*., 2012; Pastor *et al*., 2013; Firtzlaff *et al*., 2016 for drought). For this reason, the duration of the priming effect was varied in the model. To accommodate the uncertainty in parameter values, we explore the outcomes of the model under a range of parameter values.

A Euler integration method was used to simulate the dynamics over time choosing a time step of 1 h. The model was implemented in the programming language R (R Development Core Team, 2009). The model source code can be found at https://doi.org/10.5281/zenodo.838730.

### Scenarios

We explored four scenarios to analyse under which conditions priming may be beneficial. In the first scenario, we explored how the fitness benefits of priming depend on the metabolic costs of priming (*f*_prim_), the degree of light competition (α), the onset of herbivory (*t*_hs_), plant density (*D*), feeding rate (*F*_pot_), the delay in the build‐up of defense of naïve plants and the time between the priming and the trigger event. This analysis gives insight into the ecological factors that affect the benefits of priming. A benefit of priming is defined as a positive difference in seed production between the primed (*f*_prim_ > 0) and nonprimed (*f*_prim_ = 0) plants. Other parameters were kept constant.

In the second scenario, we investigated how the optimal investment in priming (i.e. the *f*_prim_ that leads to highest fitness) varies across the range of ecological conditions specified in the previous paragraph. The fitness benefit given the optimal level of priming is compared with the fitness of a focal plant that stays naïve. In addition, we tested the hypothesis that priming is a cost‐saving strategy compared with a focal plant that responds to the priming cue with inducible defense. Thus, upon reception of the priming cue, a plant induces its defense, and hence *f*_prim_ = 1. Note that this is a different comparison from our usual reference, where a focal plant stays naïve and thus only turns on its defense when attacked (*f*_prim_ = 0). If the fitness found at optimal investment in priming (*f*_prim_) is higher compared with the fitness of a focal plant that invests *f*_prim_ equal to 1, priming is more cost‐effective. In addition, we varied the costs of inducible defense (*C*_def_) and tested whether priming is more beneficial when defense costs are high. In the third scenario, we investigated the optimal level and duration of priming for different probabilities of attack, feeding rates and times of arrival. We hypothesized that, when the probability of attack is lower, the optimal duration of priming is shorter. Likewise, with lower feeding rates the optimal duration of priming is shorter. We explored two situations: (1) insect herbivores arrive with constant arrival rate, and after 15 d the cumulative probability of attack is maximal (following unpublished data from E. H. Poelman); (2) insect herbivores have a higher probability of arriving shortly after the priming cue and after 15 d the cumulative probability of attack is maximal. Finally, through applying error management theory, we explored how the decision threshold to respond to a priming cue varies with plant density and plant age. Error management theory is a decision theory that predicts that, over evolutionary time, responses will consistently be biased towards the response that avoids the most costly error. In the context of priming, two possible errors can be made: responding to the priming cue when the trigger event does not happen, or not responding to a priming cue when the trigger event does happen. The optimal response is defined by the decision threshold, which is a function of the costs of the two possible errors and the probability that a cue signals future attack. The costs entail a lower fitness compared with the right response (responding to a priming cue when the trigger event happens and not responding to a priming cue when the trigger event does not happen). The higher the decision threshold, the lower the optimal sensitivity to the priming cue (see Orrock *et al*., 2015 for details). As we have no information on the reliability of the priming cue, we assumed for these simulations that there is a 50% chance that a herbivore attack follows a priming cue, and thus the decision threshold is only influenced by size of the false positive and the false negative costs (Orrock *et al*., 2015). A global sensitivity analysis was applied using the Morris method to explore the sensitivity of the model outcomes to its input parameters (Wallach *et al*., 2014).

## Results

**Figure 3:**
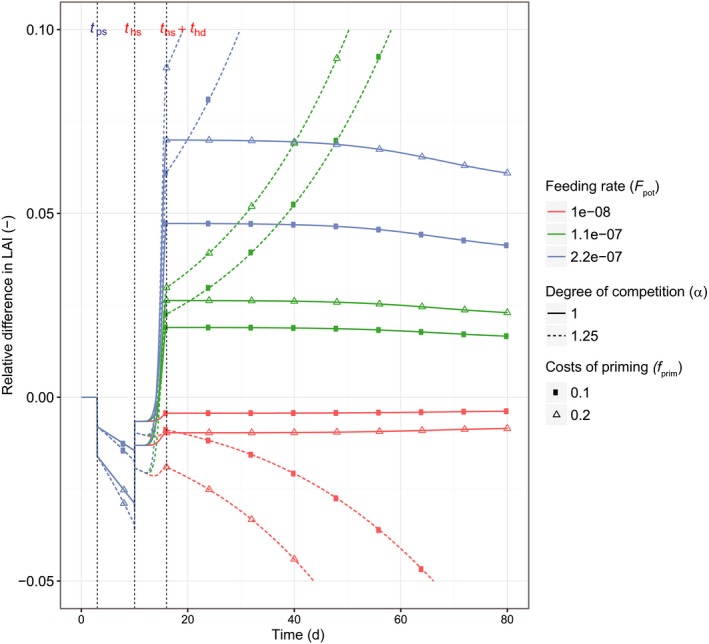
The simulated relative difference in leaf area index (LAI) over the season for a focal plant that primes for defense compared with a naïve focal plant (used here as a proxy for the relative performance value of priming). During the priming phase, the relative LAI drops as a result of investment in priming (between the onset of the priming cue (*t*_ps_) and the time at which herbivory starts (*t*_hs_)). The magnitude of the decrease depends, among other factors, on the degree of competition (α = 1, 1.25) and the costs of priming (*f*_prim_). When herbivory starts (*t*_hs_), the relative difference in LAI increases in all cases because the primed plant is initially better prepared for the attack than its naïve competitors. However, whether the benefits resulting from the investment are large enough depends, for example, on the feeding rate (*F*_pot_), the competition coefficient and the time needed for naïve competitors to reach a maximal defense level. Note that, for *t* < *t*_hs_, the red and green lines completely overlap with the blue lines. Parameter settings: number of plants per m^2^ (*D*) = 8, maintenance costs for defense against herbivores (*C*_def_) = 1, plant lifespan (*t*_s_) = 180, *t*_hs_ = 10, the time at which herbivory ends (*t*_he_) = 180, *t*_ps_ = 3, the delay in the build‐up of defensive compounds (*t*_hd_) = 6, and response pattern = earlier (Fig. 2). For other parameter settings, see Table 2.

Figure 3 illustrates the model dynamics with respect to the relative differences in LAI between the primed and the nonprimed plants until flowering (day 80; alpha = 1). At *t*_ps_, the focal priming plant primes for defense. This leads to a reduction in investment in leaf growth relative to a focal plant that does not prime. At *t*_hs_, herbivory starts, and depending on, for example, the feeding rate and the degree of light competition, the leaf area relative to its naïve competitors initially increases because the priming strategy responds faster relative to its naïve competitors. However, whether this increase can be prolonged depends on whether the benefits are sufficient to pay back the costs incurred during the priming phase, which depends, among other factors, on the feeding rate. Thus, both the costs and the benefits of priming occur through leaf area (note that we assume an insect herbivore that only eats leaves). The strength of competition (increasing α value; see (Eqn 6) had a large effect on the reduction in leaf growth during the priming phase as well as the (potential) increase in leaf growth during the trigger event, but did not have as large an effect on whether priming was beneficial or not, while the herbivore feeding rate had a large effect on whether priming was adaptive (Fig. 3). Priming was found to be beneficial when ΔLAI > 0 (difference in LAI for the priming strategy versus nonpriming) before the time at which the defense level of the nonprimed plant was maximal (*t*_hs_ + *t*_hd_).

**Figure 4:**
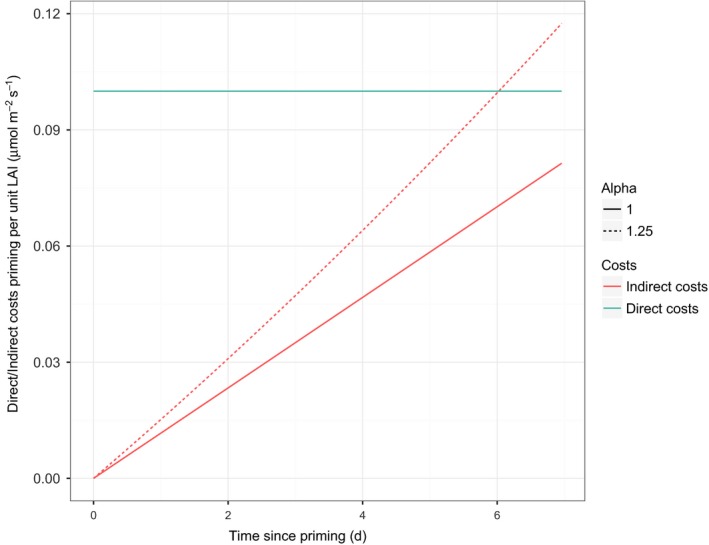
The costs of priming over time (μmol C m^−2^ s^−1^) partitioned into direct costs (per unit leaf area index (LAI); μmol C m^−2^ soil surface s^−1^; *f*_prim_) and indirect costs. Indirect costs are expressed as the reduction in net photosynthesis per unit LAI of a primed plant compared with a nonprimed plant after correcting for the reduction in net photosynthesis caused by the direct costs. Indirect costs arise because the competitors do not invest in priming and can invest in leaf growth instead, and the difference in leaf area is amplified over time. The solid line indicates symmetric competition, and the dashed line indicates asymmetric competition (the dashed line for direct costs is not visible as it is similar to the direct costs under symmetric competition). Parameter settings: number of plants per m^2^ (*D*) = 8, maintenance costs for defense against herbivores (*C*_def_) = 1, plant lifespan (*t*_s_) = 180, feeding rate (*F*_pot_) = 2.2e‐07, the time at which herbivory starts (*t*_hs_) = 10, the time at which herbivory ends (*t*_he_) = 180, the time of onset of the priming cue (*t*_ps_) = 3, the delay in the build‐up of defensive compounds (*t*_hd_) = 6, and response pattern = earlier (Fig. 2). For other parameter settings, see Table 2.

The effect of priming on leaf growth during the priming phase consisted of two components; the direct and indirect costs. The direct costs are those costs directly related to the activation and maintenance of the primed state (*f*_prim_), while the indirect costs were defined as the loss in net photosynthesis attributable to reduced leaf growth in comparison to the nonpriming strategy (resulting from the fact that direct costs entail there being less assimilates for leaf growth) until the start of herbivory (Fig. 3). The indirect costs were calculated as the difference in net photosynthesis between a primed and a nonprimed focal plant after accounting for the reduction in net photosynthesis attributable to the direct costs of priming. Thus, the indirect costs are strictly speaking a loss in income, but compared with the nonpriming strategy we consider the reduced production of assimilates a cost of the priming strategy. The indirect costs during the priming phase were found to be between 31 and 39% of the total costs during 7 d of priming and were found to increase with time (Fig. 4) and, all else being equal, to increase with time during the growing season (Fig. S1).

**Figure 5:**
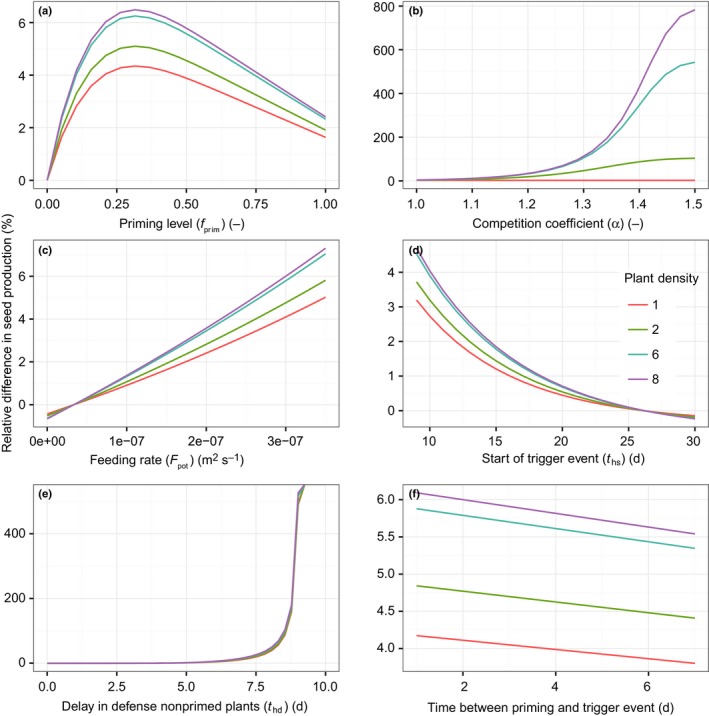
The relative change in seed production of a plant that primes for defense compared with naïve plants (used here as a proxy for the performance value of priming) as a function of (a) the direct costs of priming (*f*_prim_), (b) the competition coefficient (α), (c) the herbivore feeding rate, (d) the day during the growing season on which herbivory starts, (e) the delay in the defense response of nonprimed plants and (f) the time between the trigger and the priming event. Priming is advantageous when the relative difference in seed production is larger than zero. Parameter settings: the time at which herbivory starts (*t*_hs_) = 10, the time of onset of the priming cue (*t*_ps_) = 2, the duration of priming (*t*_pd_) = 8, α = 1, maintenance costs for defense against herbivores (*C*_def_) = 1, *f*_prim_ = 0.1, feeding rate (*F*_pot_) = 8e‐07, the delay in the build‐up of defensive compounds (*t*_hd_) = 6, log_normal_ feeding pattern, priming response pattern = earlier (Fig. 2), and flowering onset (*t*_f_) = 80. Other parameter values are listed in Table 2.

Next, we compared seed production (as a proxy for fitness) of the priming and nonpriming strategies under a range of parameter combinations. We used seed production because it integrates the benefit of a strategy over the plant's lifespan. The sensitivity analysis showed that, within the parameter ranges tested, the delay in the build‐up of defenses of the nonpriming strategy, the times when the priming and the trigger event start in the growing season, the feeding rate, and the degree of competition are the most important factors that determine whether priming is beneficial relative to the nonpriming strategy (Figs 5, S1). Priming was not found to be beneficial (thus there is lower seed production by the primed plant compared with the nonprimed plant) when the herbivore feeding rate is low (Fig. 5c), when the delay in the build‐up of defense by the nonprimed plant is short (Fig. 5e), and when priming occurs later in the growing season (Fig. 5d). These are conditions in which either the loss in leaf area as a result of herbivory or the reduction in the loss of leaf area as a result of responding faster to the trigger event is not sufficient to balance the reduced growth in leaf area during the priming phase. For the parameter values shown in Fig. 5, the direct costs of priming did not affect whether priming was beneficial or not. However, at lower feeding rates, a high investment in priming was not beneficial any more. Moreover, the relationship between *f*_prim_ and the relative fitness benefit suggests that there is an optimal investment in priming during the priming phase (Fig. 5a). In addition, the simulations show that, with increasing competition, in general the benefits of priming become larger (Fig. 5b), although at low feeding rates the disadvantage of priming may also become larger under higher competition (Fig. 3). The benefit of priming further decreases when the time between the priming and the trigger event increases, as a result of the higher costs involved in maintaining the primed state (Fig. 5f). Changing the linear relationship between the investment in defense and the reduction in feeding rate to a sigmoidal relationship (a 50% reduction in feeding rate when *C*_def_ is 0.5 and a 99% reduction in feeding rate when *C*_def_ is 1) did not change the qualitative findings, but the range under which priming was beneficial was reduced. In particular, the feeding rate and the degree of competition had a larger effect on determining whether priming was beneficial (Fig. S3).

**Figure 6:**
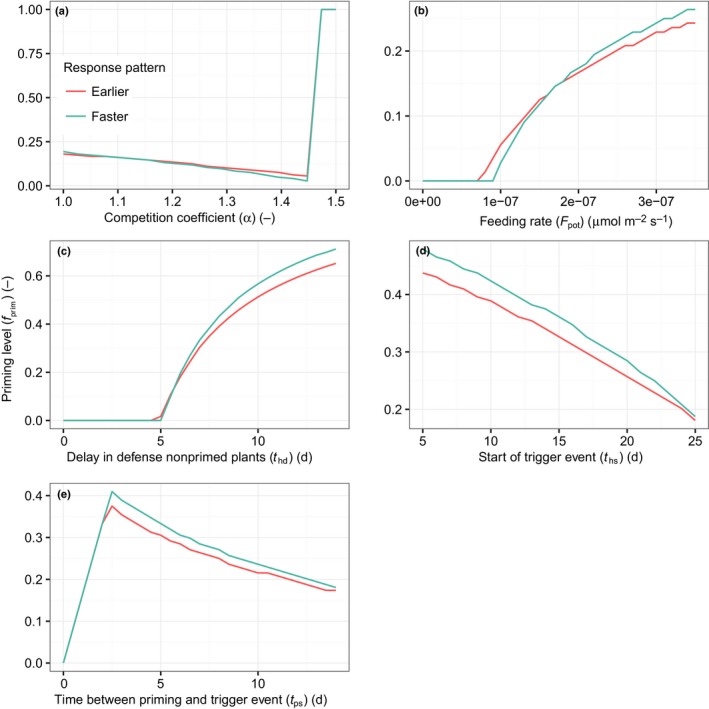
The level of priming (*f*_prim_) that leads to highest fitness for two different response types (red, earlier; blue, faster) as a function of (a) the competition coefficient (α), (b) the feeding rate, (c) the delay in the defense of nonprimed plants, (d) the start of the trigger event during the season, and (e) the time between the priming and the trigger event. When the optimal level is equal to zero it indicates that it is not beneficial to prime. For each panel, default parameter values were chosen and the one depicted changed over its range. Parameter values: the time at which herbivory starts (*t*_hs_) = 15, the time of onset of the priming cue (*t*_ps_) = 2, the duration of priming (*t*_pd_) = 13, α = 1, maintenance costs for defense against herbivores (*C*_def_) = 1, *f*_prim_ = 0.1, feeding rate (*F*_pot_) = 2.2e‐07, the delay in the build‐up of defensive compounds (*t*_hd_) = 6, log_normal_ feeding pattern, priming response pattern = earlier (Fig. 2), and flowering onset (*t*_f_) = 80. Other parameter values are listed in Table 2.

When plants are grown in competition with naïve plants, the optimal investment in priming depends on the growing conditions (Fig. 6). The optimal investment results from the balance between the advantage of responding faster to the trigger event, and thus avoiding damage, and the costs that are paid during the priming phase (to avoid the damage later). For example, when competition for light is stronger, leaf growth during the priming phase is more reduced. Hence, the optimal investment in priming decreases (Fig. 6a). However, when competition for light becomes very severe, the ecological costs of priming become very large and to compensate for these costs a fast response to the trigger event is needed. In the same way, when the time between the priming cue and attack increases, the total time over which costs are paid increases, while the benefits remain equal (Fig. 6e). Hence, a lower investment in priming is optimal. When priming occurs later in the growing season, the optimal investment in priming decreases (Fig. 6d). This is because, in denser canopies, the net photosynthesis per unit leaf area is lower while the costs of priming per unit leaf area remain constant. Furthermore, the optimal investment in priming increases with feeding rate and with an increasing delay experienced by naïve plants in building up their defenses (Fig. 6b,c). This is because, in those conditions, naïve plants suffer more damage when attacked and the primed plant can afford to invest more in priming. The optimal level of investment in priming was in almost all cases < 1, which suggests that priming in response to a priming cue is more beneficial compared with a strategy that induces defense in response to the priming cue.

Three different priming response patterns have been distinguished in the literature (Fig. 2). We tested these response patterns in the model to explore the ecological conditions that may favor each of the responses. The type of response pattern of the primed plant, be it responding faster or earlier, only had a small effect on the optimal level of priming, in a complex fashion. On average, the faster response had a higher optimal investment in priming compared with the earlier response. The ‘earlier’ priming strategy was beneficial at relatively low feeding rates, while the ‘faster’ priming strategy was not. This is because the ‘earlier’ priming strategy starts at higher defense levels when the trigger event occurs compared with the faster response. At intermediate feeding rates, and when the degree of competition was relatively high, the faster response had lower optimal priming levels compared with the earlier priming response (Fig. 6a,b). Hence plants with the latter response pattern achieved maximum fitness with lower investment in priming compared to the strategy that responds faster. The priming response type ‘stronger’ – a priming strategy that entailed more investment in defense compared with naïve plants – was only found to be beneficial if the investment in defense of naïve plants was suboptimal for the experienced herbivore feeding rate (Fig. S4).

**Figure 7:**
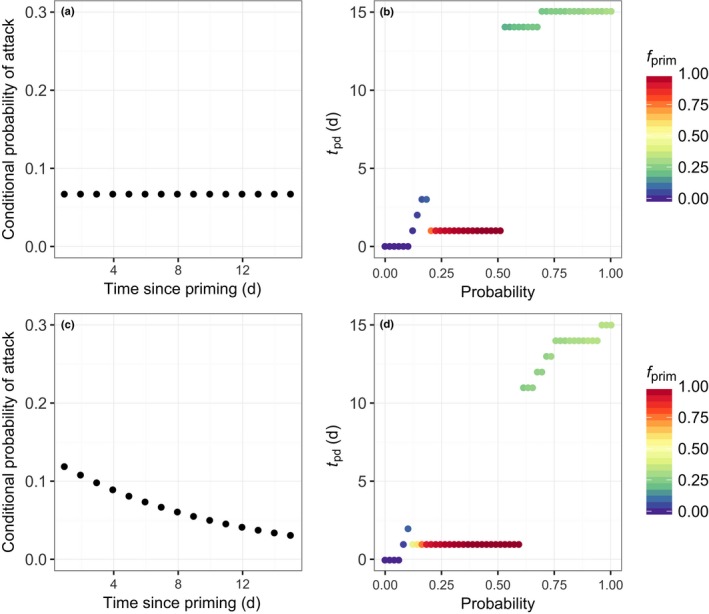
The combined optimal duration of priming (*t*_pd_; b, d) and the level of priming (*f*_prim_; color coding in those panels) as a function of the probability of attack for two arrival patterns. In the first scenario (a), it is assumed that if that attack occurs, this probability is constant over time. The second scenario (c) assumes that if the attack occurs, this probability decreases with time. Following field observations, the sum of the conditional probability of attack is 1 after 15 d. The costs of priming (*f*_prim_) that leads to maximum fitness is indicated by the colors.

We further explored how the optimal duration over which plants stay primed varied with the probability of attack and the arrival dynamics of the herbivores (Fig. 7). With increasing probability of attack, both the optimal duration of priming and the optimal investment (*f*_prim_) increase. At low probabilities of attack, low investment over a short duration gives the highest fitness. In this situation, it is unlikely that the trigger will occur, and hence a low investment in priming for a short period of time is optimal. At intermediate probabilities of attack, high but short investment in priming is preferred over low but long investment in priming (Fig. 7b,d). At high probabilities of attack, prolonged, low‐level investment in priming is optimal. Because it is very likely that the attack will occur, and thus that the plant gets a return on investment somewhere within the next 15 d, it is favorable to stay primed for this time period. However, because the priming costs are paid over an, on average, longer time span this reduces the optimal investment in priming (Fig. 6e).

Application of error management theory to analyze the optimal response to an uncertain priming cue showed that the sensitivity to a cue of a given reliability should decrease with plant age, and the time in the growing season when priming occurs (Fig. S5), because the costs of priming when the herbivore attack does not occur are larger compared with the costs of not priming when the herbivore does show up. When the feeding rate is high, less evidence is needed that the priming cue signals future herbivore attack.

## Discussion

**Figure 8:**
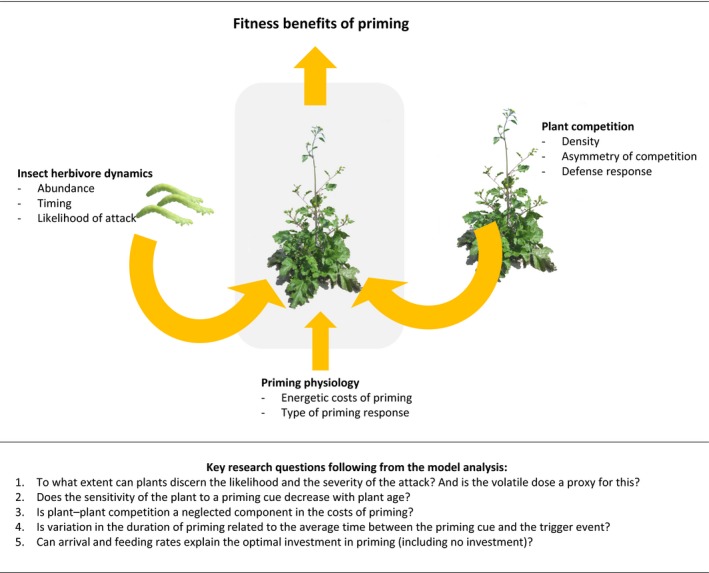
A graphical summary of this study. The model analysis shows that the adaptive benefit of priming depends on the community context, for example plant–plant competition and insect herbivore dynamics, the priming physiology and their interplay. By explicitly considering the community context, a number of key questions arise.

While understanding the mechanisms of priming and their associated costs is an active field of research, the question of how the ecological context shapes the costs and benefits of priming has, so far, received little attention. Our model explores how priming physiology and biotic components, such as feeding and arrival rates of the insect herbivores, shape the costs and benefits of priming. The modeling analysis suggests that the adaptive value of priming is strongly affected by the biotic environment and to a lesser extent by the physiology of the primed plant (see Fig. 8 for a graphical summary).

### The benefits of priming are altered through the competitive environment

The fitness benefits of priming result from the balance between the investment in priming and the return on investment. In the model, we assumed that a primed plant starts incurring priming costs after the priming event has taken place, while the return on investment only occurs if/after the trigger event has taken place. The direct (energetic) costs are affected by the priming physiology: the investment in priming per unit time (*f*_prim_) and the duration of priming (*t*_pd_). The indirect costs were affected by these direct costs and the degree to which competition for light is asymmetric (i.e. the α value). Indirect costs increase over time because photosynthates that were used for priming could alternatively have been spent on leaf growth and this reduces net photosynthesis and thus additional growth relative to the nonprimed plant (i.e. a loss in income). Studies that estimated the costs of priming did not grow plants in competition (van Hulten *et al*., 2006; Walters *et al*., 2008) and hence these indirect costs were not included in their estimates of the costs of priming. Yet, the model results suggest that under competition the indirect costs of priming can be quite substantial. They were estimated to be between 31 and 39% of the total priming costs (calculated over 7 d of priming; Fig. 3) and these costs increase when competition for light increases. In a community context, the priming cue may be spatially heterogeneous, leading to a heterogeneous pattern of primed and nonprimed plants which may, depending on the extent to which priming is cue dose‐dependent, result in different levels of priming among plants and thus in substantial indirect costs for the primed plants (Karban *et al*., 2003; Heil & Adame‐Álvarez, 2010). The model results therefore suggest, in line with findings of Rillig *et al*. (2015), that competition is a key factor that should be included when studying the adaptiveness of priming.

### Interactions with insect herbivores and neighboring plants shape the benefits of priming

In the model, we assumed that the priming strategy may pay off only after the trigger event has taken place, because a primed plant increases its defenses faster upon the trigger event and thus initially suffers from lower levels of herbivory and associated losses in LAI than a nonprimed plant. Whether this reduced loss in LAI compensates for the reduced growth during the priming phase depends on the interplay of physiological factors, such as the response pattern of the primed plant (earlier or faster priming) and biotic factors, such as the feeding rate, the degree of competition between plants, the delay that naïve plants experience in building up their defenses (first scenario), and the probability of attack (third scenario). We found relatively small differences between the ‘early’ and ‘faster’ priming strategies. By contrast, a ‘stronger’ response to the trigger event could help a plant survive if it was suboptimally defended otherwise. Most of the factors that determined the benefits of priming, such as the feeding rate and the probability of attack, are beyond the plant's control. Therefore, we expect that priming is more likely to have evolved in environments where the feeding rate is high, the time between the priming and the trigger event is short and the probability of attack is high. Indeed, Walters *et al*. (2008) showed that priming was beneficial under high disease pressure only. In summary, we found that the feeding rate, the arrival rate and the probability of arrival have a large effect on the benefits of and the optimal investment in priming. However, to our knowledge, arrival rates and the probability of attack on wild species are rarely reported in the literature. Thus, our findings call for controlled experiments that vary arrival and feeding rates relative to those observed in the field to elucidate how differences in the biotic environment affect the benefits of priming.

### The optimal investment in priming is affected by the likelihood of attack, the timing of attack, and the severity of attack

Priming has been suggested to have evolved as a strategy to avoid the full costs of induced resistance in response to a priming cue (Frost *et al*., 2008). This hypothesis was confirmed by the model simulations: in nearly all cases (except when the degree of competition was very strong) priming led to a higher fitness compared with inducing defense in response to a priming cue (Fig. 6). This advantage became larger when the costs of defense increased (Fig. S6).

The model results further show that the likelihood of attack, the timing of attack, and the severity of attack affect the optimal investment in priming (scenarios 2 and 3; Figs 6, 7). This implies that the adaptive benefit of priming may increase if such information can be derived from the priming cue. Whether or how plants do this is still unknown. The volatile dose could be a good candidate to contain information on the probability and severity of the upcoming attack because it was found to be related to the amount of leaf damage (Niinemets *et al*., 2013; Shiojiri *et al*., 2010; Clavijo McCormick *et al*., 2014). Moreover, there is indirect evidence that plants respond to volatiles in a dose‐dependent manner (Dolch & Tscharntke, 2000), although this has never been shown directly (Karban *et al*., 2006; Heil & Adame‐Álvarez, 2010). The sensitivity to the priming cue may depend on whether receiver plants share the herbivore community that feeds on the emitter plant. For herbivore‐induced plant volatiles (HIPVs), Karban *et al*. (2013a) showed that neighboring plants respond more strongly to HIPVs of genetically more closely related individuals than to HIPVs of individuals that are less closely related, and Karban *et al*. (2004) showed that not all species are equally responsive to volatiles produced by sagebrush (*Artemisia tridentata*).

### The optimal duration of priming follows the dynamics of insect herbivore attack

The optimal duration of the primed state is, like the optimal level of priming, positively related to the feeding rate and the probability of attack, and negatively related to the time between the priming cue and the trigger event. We are not aware of any study on variation in the duration of priming for defense in relation to the average time between the priming cue and the trigger event, but studies on vernalization – that is, the acceleration of flowering after prolonged cold – show that accessions from northern regions require longer cold periods compared with accessions from southern regions before showing a vernalization response (Shindo *et al*., 2006; Song *et al*., 2013). Thus, it should be investigated if the time over which the plant stays primed affects the pay‐off of priming. In the same way, studies contrasting accessions from areas with different herbivore arrival dynamics may help to elucidate the time during which plants stay primed. Experiments on annual plants showed that the primed state can range from one to several days (Ding *et al*., 2012; Ali *et al*., 2013; Firtzlaff *et al*., 2016). There may be three explanations for this relatively short duration. First, the trigger event for which the primed state prepares is most likely to occur within days after the priming event. Indeed, the model analysis showed that if the probability of attack is higher shortly after the priming cue, on average the optimal duration of the primed state will decrease. Second, according to the model, a short duration of the primed state is optimal when it is highly uncertain whether the trigger event will occur. This finding relies on the critical assumption that priming costs are paid as long as the plant stays primed. Whether this is a valid assumption remains to be investigated, as one could assume alternatively that priming requires relatively high investment costs and lower maintenance costs (Martinez‐Medina *et al*., 2016). Finally, the priming response to a particular stressor may prevent or delay the response to another stressor (so called *trans*‐priming; Hilker *et al*., 2015). For example, infection with pathogens may decrease resistance to herbivores as a consequence of the cross‐talk between the jasmonic acid and salicylic acid pathways (Felton & Korth, 2000). In the case of such a trade‐off, the ecological costs of priming may be substantial when the plant is prepared for the wrong attacker, even when the metabolic costs of priming are zero (Crisp *et al*., 2016).

### The optimal priming strategy under uncertainty of insect herbivore attack

So far, we have assumed that the priming cue is a reliable predictor of the trigger event. In reality, a plant needs to discern noise from the true signal (Karban *et al*., 1999). When the cue is not a reliable indicator of the trigger event, two wrong choices can be made: responding to a cue that does not signal the trigger event and not responding to a cue that does signal the trigger event. Error management theory predicts that, over evolutionary time, plant responses will be biased towards the choice that incurs the least costs (Haselton & Nettle, 2006; Orrock *et al*., 2015). When this was applied to the priming responses of young and older plants (in the model we varied the timing of the priming and the trigger event in the season), the model results showed that older plants were predicted to be less responsive to a priming cue of given strength than younger plants, consistent with the finding of Shiojiri & Karban (2006), and this effect was more pronounced when competition for light was stronger. Plants that are primed later in the growing season benefit less from priming for at least two reasons. First, the proportion of leaf area consumed early in the season is larger than that consumed later in the season, when plants have more leaf area. Hence, the benefit of reducing the feeding rate through defense is larger early in the growing season. Second, the relative costs of priming expressed per unit leaf area increase during the growing season because the amount of gross photosynthesis generated per unit leaf area decreases as a result of (self‐)shading, while the metabolic costs of priming per unit leaf area remain constant.

The finding that, given its benefits, the priming strategy is likely to evolve in environments that experience high feeding rates and where the priming cue is a reliable predictor of the trigger event happening hinges on a number of assumptions. Some are related to the physiology of priming, while others are related to the ecological context in which priming occurs. Regarding the former, it is a big unknown how the direct priming costs relate to the level at which the plant is primed (at the DNA level, at the metabolic level or at higher levels) and how this translates to a modified response to the trigger event. In this model, we assumed – given a lack of evidence of more complex functional forms – a linear relationship between the investment in priming and the rate at which defenses were built up. However, the benefits of priming will increase when a low investment in priming gives a relatively higher reduction in feeding rate. Moreover, the benefits of priming would increase when the time during which priming costs are paid is short while the benefits last longer. Finally, resource allocation to roots was assumed to be constant in the model, while it has been reported that herbivory may lead to increased allocation to roots (Schwachtje *et al*., 2006). Moreover, recent evidence showed that HIPVs may increase root development in receiver plants as well (Sweeney *et al*., 2017). Thus, how those two processes play out in their effects on fitness is as yet unknown.

### Conclusions and future outlook

This study showed that putting priming in a community context affects the conditions in which priming is adaptive. By explicitly considering the ecological context of priming, this study raises a number of key questions for future research (see also Fig. 8), How do the benefits of priming change when plant–plant competition is included? Do plants become less priming‐responsive with age? To what extent can plants discern the probability and timing of trigger events from priming cues? Can herbivore arrival dynamics explain the investment in priming and the time over which plants stay primed? We conclude that a great leap in understanding the evolution of priming can be made when plant‐plant competition and herbivore dynamics are taken into account.

## Author contributions

J.C.D., E.H.P., M.D. and N.P.R.A. designed the research. J.C.D. and P.J.V. developed the model and J.C.D. performed the simulations. J.C.D., P.J.V., E.H.P., M.D. and N.P.R.A. wrote the manuscript.

## Supporting information

Please note: Wiley Blackwell are not responsible for the content or functionality of any Supporting Information supplied by the authors. Any queries (other than missing material) should be directed to the *New Phytologist* Central Office.

**Fig. S1** Relationship between the onset of herbivory and the benefits of priming assuming the feeding rate is proportional to leaf area.
**Fig. S2** Results of the sensitivity analysis.
**Fig. S3** The benefit of priming assuming a sigmoidal relationship between investment in defense and reduction in feeding rate.
**Fig. S4** Fitness benefits of priming when assuming different priming response patterns.
**Fig. S5** The relationship between onset of herbivory and the decision threshold.
**Fig. S6** The relationship between the costs of defense and the benefit of priming.
**Table S1** Overview of the most important model assumptions

## References

[nph14771-bib-0001] Ali M , Sugimoto K , Ramadan A , Arimura G‐I . 2013 Memory of plant communications for priming anti‐herbivore responses. Scientific Reports 3: 1872. 23695148 10.1038/srep01872 PMC3660719

[nph14771-bib-0002] Bruce TJA , Matthes MC , Napier JA , Pickett JA . 2007 Stressful “memories” of plants: evidence and possible mechanisms. Plant Science 173: 603–608.

[nph14771-bib-0003] Clavijo McCormick A , Boeckler GA , Köllner TG , Gershenzon J , Unsicker SB . 2014 The timing of herbivore‐induced volatile emission in black poplar (*Populus nigra*) and the influence of herbivore age and identity affect the value of individual volatiles as cues for herbivore enemies. BMC Plant Biology 14: 304. 25429804 10.1186/s12870-014-0304-5 PMC4262996

[nph14771-bib-0004] Conrath U , Beckers GJM , Flors V , Garcia‐Agustin P , Jakab G , Mauch F , Newman MA , Pieterse CMJ , Poinssot B , Pozo MJ *et al* 2006 Priming: getting ready for battle. Molecular Plant–Microbe Interactions 19: 1062–1071. 17022170 10.1094/MPMI-19-1062

[nph14771-bib-0005] Conrath U , Beckers GJM , Langenbach CJG , Jaskiewicz MR . 2015 Priming for enhanced defense. Annual Review of Phytopathology 53: 97–119. 10.1146/annurev-phyto-080614-120132 26070330

[nph14771-bib-0006] Crisp PA , Ganguly D , Eichten SR , Borevitz JO , Pogson BJ . 2016 Reconsidering plant memory: intersections between stress recovery, RNA turnover, and epigenetics. Science Advances 2: e1501340. 26989783 10.1126/sciadv.1501340 PMC4788475

[nph14771-bib-0007] DeWitt TJ , Sih A , Wilson DS . 1998 Costs and limits of phenotypic plasticity. Trends in Ecology & Evolution 13: 77–81. 21238209 10.1016/s0169-5347(97)01274-3

[nph14771-bib-0008] Dicke M , van Loon JJA , Soler R . 2009 Chemical complexity of volatiles from plants induced by multiple attack. Nature Chemical Biology 5: 317–324. 19377458 10.1038/nchembio.169

[nph14771-bib-0009] Ding Y , Fromm M , Avramova Z . 2012 Multiple exposures to drought ‘train’ transcriptional responses in Arabidopsis. Nature Communications 3: 740. 10.1038/ncomms1732 22415831

[nph14771-bib-0010] Dolch R , Tscharntke T . 2000 Defoliation of alders (*Alnus glutinosa*) affects herbivory by leaf beetles on undamaged neighbours. Oecologia 125: 504–511. 28547220 10.1007/s004420000482

[nph14771-bib-0011] Felton GW , Korth KL . 2000 Trade‐offs between pathogen and herbivore resistance. Current Opinion in Plant Biology 3: 309–314. 10873851 10.1016/s1369-5266(00)00086-8

[nph14771-bib-0012] Firtzlaff V , Oberländer J , Geiselhardt S , Hilker M , Kunze R . 2016 Pre‐exposure of Arabidopsis to the abiotic or biotic environmental stimuli “chilling” or “insect eggs” exhibits different transcriptomic responses to herbivory. Scientific Reports 6: 28544. 27329974 10.1038/srep28544 PMC4916510

[nph14771-bib-0013] Frost CJ , Appel M , Carlson JE , De Moraes CM , Mescher MC , Schultz JC . 2007 Within‐plant signalling via volatiles overcomes vascular constraints on systemic signalling and primes responses against herbivores. Ecology Letters 10: 490–498. 17498148 10.1111/j.1461-0248.2007.01043.x

[nph14771-bib-0014] Frost CJ , Mescher MC , Carlson JE , De Moraes CM . 2008 Plant defense priming against herbivores: getting ready for a different battle. Plant Physiology 146: 818–824. 18316635 10.1104/pp.107.113027 PMC2259053

[nph14771-bib-0015] Haselton MG , Nettle D . 2006 The paranoid optimist: an integrative evolutionary model of cognitive biases. Personality and Social Psychology Review 10: 47–66. 16430328 10.1207/s15327957pspr1001_3

[nph14771-bib-0016] Heil M , Adame‐Álvarez RM . 2010 Short signalling distances make plant communication a soliloquy. Biology Letters 6: 843–845. 20554558 10.1098/rsbl.2010.0440 PMC3001381

[nph14771-bib-0017] Heil M , Silva Bueno JC . 2007 Within‐plant signaling by volatiles leads to induction and priming of an indirect plant defense in nature. Proceedings of the National Academy of Sciences, USA 104: 5467–5472. 10.1073/pnas.0610266104 PMC1838500 17360371

[nph14771-bib-0018] Heil M , Ton J . 2008 Long‐distance signalling in plant defence. Trends in Plant Science 13: 264–272. 18487073 10.1016/j.tplants.2008.03.005

[nph14771-bib-0019] Hilker M , Schwachtje J , Baier M , Balazadeh S , Bäurle I , Geiselhardt S , Hincha DK , Kunze R , Mueller‐Roeber B , Rillig MC *et al* 2015 Priming and memory of stress responses in organisms lacking a nervous system. Biological Reviews 91: 1118–1133. 26289992 10.1111/brv.12215

[nph14771-bib-0020] van Hulten M , Pelser M , van Loon LC , Pieterse CMJ , Ton J . 2006 Costs and benefits of priming for defense in Arabidopsis. Proceedings of the National Academy of Sciences, USA 103: 5602–5607. 10.1073/pnas.0510213103 PMC1459400 16565218

[nph14771-bib-0021] Jakab G , Ton J , Flors V , Zimmerli L , Métraux J‐P , Mauch‐Mani B . 2005 Enhancing Arabidopsis salt and drought stress tolerance by chemical priming for its abscisic acid responses. Plant Physiology 139: 267–274. 16113213 10.1104/pp.105.065698 PMC1203376

[nph14771-bib-0022] Karban R , Agrawal AA , Thaler JS , Adler LS . 1999 Induced plant responses and information content about risk of herbivory. Trends in Ecology & Evolution 14: 443–447. 10511721 10.1016/s0169-5347(99)01678-x

[nph14771-bib-0023] Karban R , Huntzinger M , McCall AC . 2004 The specificity of eavesdropping on sagebrush by other plants. Ecology 85: 1846–1852.

[nph14771-bib-0024] Karban R , Maron J , Felton GW , Ervin G , Eichenseer H . 2003 Herbivore damage to sagebrush induces resistance in wild tobacco: evidence for eavesdropping between plants. Oikos 100: 325–332.

[nph14771-bib-0205] Karban R , Shiojiri K . 2009 Self‐recognition affects plant communication and defense. Ecology Letters 12: 502‐506. 19392712 10.1111/j.1461-0248.2009.01313.x

[nph14771-bib-0025] Karban R , Shiojiri K , Huntzinger M , McCall AC . 2006 Damage‐induced resistance in sagebrush: volatiles are key to intra‐ and interplant communication. Ecology 87: 922–930. 16676536 10.1890/0012-9658(2006)87[922:drisva]2.0.co;2

[nph14771-bib-0026] Karban R , Shiojiri K , Ishizaki S , Wetzel WC , Evans RY . 2013a Kin recognition affects plant communication and defence. Proceedings of the Royal Society B: Biological Sciences 280: 20123062. 23407838 10.1098/rspb.2012.3062 PMC3574382

[nph14771-bib-0027] Karban R , Yang LH , Edwards KF . 2013b Volatile communication between plants that affects herbivory: a meta‐analysis. Ecology Letters 17: 44–52. 24165497 10.1111/ele.12205

[nph14771-bib-0028] Kessler A , Halitschke R , Diezel C , Baldwin IT . 2006 Priming of plant defense responses in nature by airborne signaling between *Artemisia tridentata* and *Nicotiana attenuata* . Oecologia 148: 280–292. 16463175 10.1007/s00442-006-0365-8

[nph14771-bib-0029] Kim J , Felton GW . 2013 Priming of antiherbivore defensive responses in plants. Insect Science 20: 273–285. 23955880 10.1111/j.1744-7917.2012.01584.x

[nph14771-bib-0030] León J , Rojo E , Sánchez‐Serrano JJ . 2001 Wound signalling in plants. Journal of Experimental Botany 52: 1–9. 10.1093/jexbot/52.354.1 11181708

[nph14771-bib-0031] Martinez‐Medina A , Flors V , Heil M , Mauch‐Mani B , Pieterse CM , Pozo MJ , Ton J , van Dam NM , Conrath U . 2016 Recognizing plant defense priming. Trends in Plant Science 21: 818–822. 27507609 10.1016/j.tplants.2016.07.009

[nph14771-bib-0032] Morrell K , Kessler A . 2016 Plant communication in a widespread goldenrod: keeping herbivores on the move. Functional Ecology 31: 1049–1061.

[nph14771-bib-0033] Niinemets Ü , Kännaste A , Copolovici L . 2013 Quantitative patterns between plant volatile emissions induced by biotic stresses and the degree of damage. Frontiers in Plant Science 4: 262. 23888161 10.3389/fpls.2013.00262 PMC3719043

[nph14771-bib-0034] Orrock JL , Sih A , Ferrari MCO , Karban R , Preisser EL , Sheriff MJ , Thaler JS . 2015 Error management in plant allocation to herbivore defense. Trends in Ecology & Evolution 30: 441–445. 26138386 10.1016/j.tree.2015.06.005

[nph14771-bib-0035] Pastor V , Luna E , Mauch‐Mani B , Ton J , Flors V . 2013 Primed plants do not forget. Environmental and Experimental Botany 94: 46–56.

[nph14771-bib-0036] Peng J , van Loon JJA , Zheng S , Dicke M . 2011 Herbivore‐induced volatiles of cabbage (*Brassica oleracea*) prime defence responses in neighbouring intact plants. Plant Biology 13: 276–284. 21309974 10.1111/j.1438-8677.2010.00364.x

[nph14771-bib-0037] R Development Core Team . 2009 R: a language and environment for statistical computing, v.3.4.0. Vienna, Austria: R Foundation for Statistical Computing ISBN 3‐900051‐07‐0. [WWW document] URL http://www.R-project.org.

[nph14771-bib-0038] Rillig MC , Rolff J , Tietjen B , Wehner J , Andrade‐Linares DR . 2015 Community priming – effects of sequential stressors on microbial assemblages. FEMS Microbiology Ecology 91: fiv040. 25873462 10.1093/femsec/fiv040

[nph14771-bib-0039] Rodriguez‐Saona CR , Rodriguez‐Saona LE , Frost CJ . 2009 Herbivore‐induced volatiles in the perennial shrub, *Vaccinium corymbosum*, and their role in inter‐branch signaling. Journal of Chemical Ecology 35: 163–175. 19159981 10.1007/s10886-008-9579-z

[nph14771-bib-0040] Schmitt J , Stinchcombe JR , Heschel MS , Huber H . 2003 The adaptive evolution of plasticity: phytochrome‐mediated shade avoidance responses. Integrative and Comparative Biology 43: 459–469. 21680454 10.1093/icb/43.3.459

[nph14771-bib-0041] Schwachtje J , Minchin PEH , Jahnke S , van Dongen JT , Schittko U , Baldwin IT . 2006 SNF1‐related kinases allow plants to tolerate herbivory by allocating carbon to roots. Proceedings of the National Academy of Sciences, USA 103: 12935–12940. 10.1073/pnas.0602316103 PMC1568949 16912118

[nph14771-bib-0042] Shindo C , Lister C , Crevillen P , Nordborg M , Dean C . 2006 Variation in the epigenetic silencing of FLC contributes to natural variation in Arabidopsis vernalization response. Genes & Development 20: 3079–3083. 17114581 10.1101/gad.405306 PMC1635143

[nph14771-bib-0043] Shiojiri K , Karban R . 2006 Plant age, communication, and resistance to herbivores: young sagebrush plants are better emitters and receivers. Oecologia 149: 214–220. 16736187 10.1007/s00442-006-0441-0

[nph14771-bib-0044] Shiojiri K , Ozawa R , Kugimiya S , Uefune M , van Wijk M , Sabelis MW , Takabayashi J . 2010 Herbivore‐specific, density‐dependent induction of plant volatiles: honest or “cry wolf” signals? PLoS ONE 5: e12161. 20808961 10.1371/journal.pone.0012161 PMC2923144

[nph14771-bib-0045] Song J , Irwin J , Dean C . 2013 Remembering the prolonged cold of winter. Current Biology 23: R807–R811. 24028964 10.1016/j.cub.2013.07.027

[nph14771-bib-0046] Stam JM , Kroes A , Li Y , Gols R , van Loon JJ , Poelman EH , Dicke M . 2014 Plant interactions with multiple insect herbivores: from community to genes. Annual Review of Plant Biology 65: 689–713. 10.1146/annurev-arplant-050213-035937 24313843

[nph14771-bib-0047] Sweeney C , Lakshmanan V , Bais HP . 2017 Interplant aboveground signaling prompts upregulation of auxin promoter and malate transporter as part of defensive response in the neighboring plants. Frontiers in Plant Science 8: 595. 28469632 10.3389/fpls.2017.00595 PMC5395557

[nph14771-bib-0048] Ton J , D'Alessandro M , Jourdie V , Jakab G , Karlen D , Held M , Mauch‐Mani B , Turlings TCJ . 2007 Priming by airborne signals boosts direct and indirect resistance in maize. Plant Journal 49: 16–26. 17144894 10.1111/j.1365-313X.2006.02935.x

[nph14771-bib-0049] Vermeulen PJC . 2015 On selection for flowering time plasticity in response to density. New Phytologist 205: 429–439. 25124368 10.1111/nph.12984

[nph14771-bib-0050] Wallach D , Makowski D , Jones JW , Brun F . 2014 Working with dynamic crop models – methods, tools, and examples for agriculture and environment. London, UK: Elsevier.

[nph14771-bib-0051] Walters DR , Paterson L , Walsh DJ , Havis ND . 2008 Priming for plant defense in barley provides benefits only under high disease pressure. Physiological and Molecular Plant Pathology 73: 95–100.

