# Supplementary material for: When does it pay off to prime for defense? A modeling analysis
Source: New Phytol. 2017 Sep 11;216(3):782–97. doi: 10.1111/nph.14771 (PMC5659137; doi:10.1111/nph.14771)
Supplement: Supplementary file 1 — Fig. S1 Relationship between the onset of herbivory and the benefits of priming assuming the feeding rate is proportional to leaf area. Fig. S2 Results of the sensitivity analysis. Fig. S3 The benefit of priming assuming a sigmoidal relationship between investment in defense and reduction in feeding rate. Fig. S4 Fitness benefits of priming when assuming different priming response patterns. Fig. S5 The relationship between onset of herbivory and the decision threshold. Fig. S6 The relationship between the costs of defense and the benefit of priming. Table S1 Overview of the most important model assumptions [file NPH-216-782-s001.pdf]

New Phytologist Supporting Information Figs S1-S6 and Table S1

Article Title: When does it pay-off to prime for defense? A model analysis

Authors: Jacob C. Douma, Peter J. Vermeulen, Erik H. Poelman, Marcel Dicke, Niels P.R. Anten

Article acceptance data: 30 July 2017

Table S1 Overview of the most important model assumptions

Model Component	Assumption
Volatiles	All plants receive the cue, but only a plant that is able to prime moves into a primed state when receiving the cue.
	Volatile concentration is constant. The reliability of the cue is incorporated in the probability to be attacked. The latter was varied in the model
Insect herbivores	Leaf herbivory follows a lognormal pattern over time (following unpublished data of E. Poelman), with a peak at 32 days.
	An increased reduction in feeding rate does not lead to increased movement of the insect herbivore.
	Insect herbivores consume leafs only.
	The feeding rate is expressed per plant. Thus the amount of leaf area removed/m2 soil surface increases with plant density.
Plant defense:	There is linear relationship between the investment in defense and reduction in feeding rate. 100% reduction of feeding rate is possible (representing that insect herbivores move away from the defended plant). We have played with a sigmoidal relationship between defense and the reduction in feeding rate, but the results did not change qualitatively (data not shown).
	Defense costs are paid as maintenance costs and not as construction costs.
	Plants experience a delay before defense is at its desired level. The delay is a parameter that was varied in the model
	A primed plant does experience a delay that is shorter than the non-primed plant. The length of this delay depends on its investment in priming. Investment in priming is a parameter that was varied in the model

Plant priming:	Priming costs are paid as maintenance costs. Investment in priming is a parameter that was varied in the model<br><br>As long as priming costs are paid, priming is effective. When priming costs are not paid anymore, the priming effect vanishes at the same speed as that it took to build up the priming effect.<br><br>There is some time between the priming event and the actual attack (a parameter that is varied in the model). The time between the priming cue and the attack was varied in the model.
Plant growth	Plants are annuals<br><br>Plants do not have (storage) organs from which carbon can be allocated after/during herbivory<br><br>At flowering time, plants completely switch from the vegetative to the regenerative phase.<br><br>Plant experience optimal growing conditions, temperature does not affect growth.

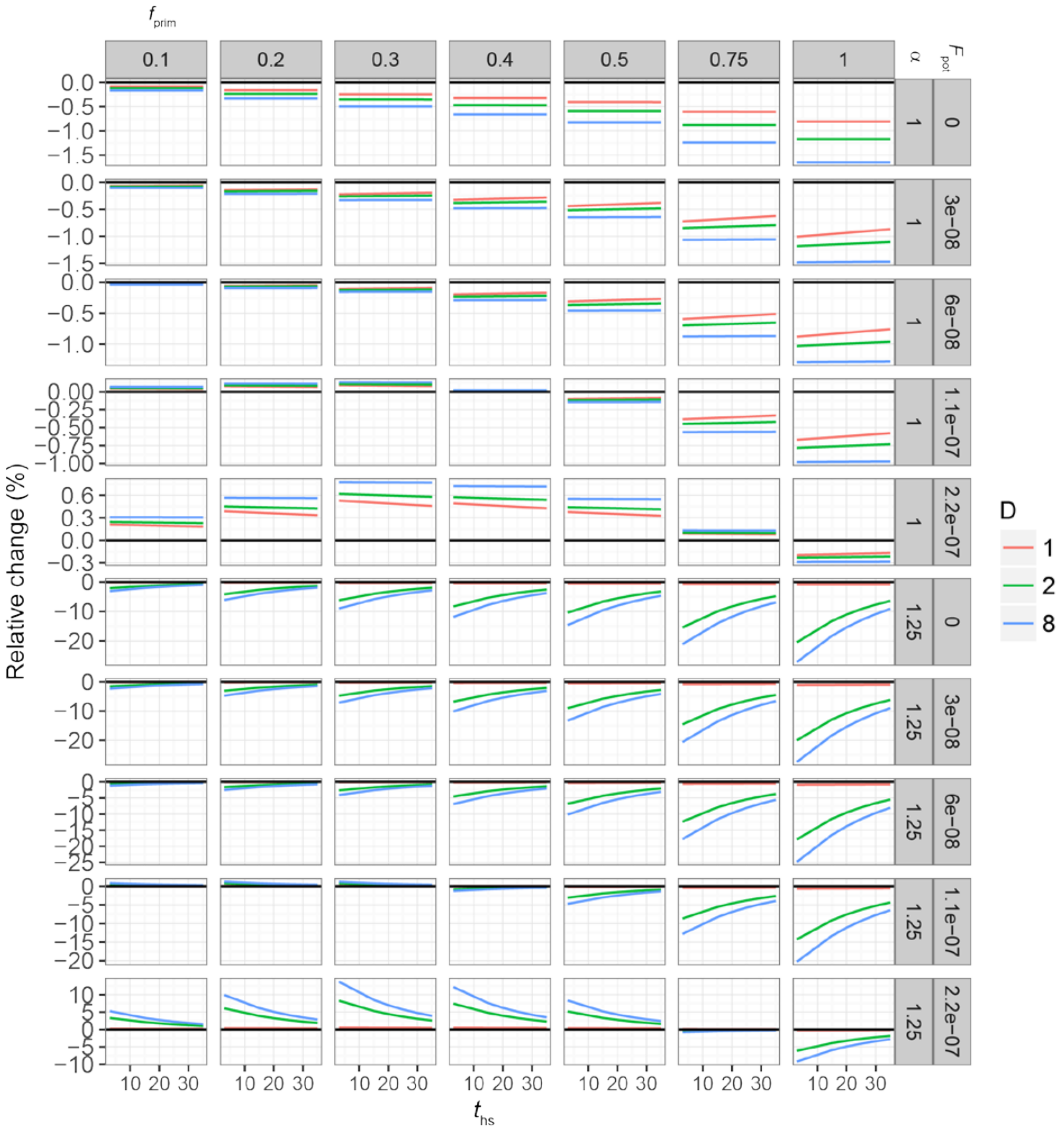

Fig. S1. Relationship between the onset of herbivory and the relative costs and benefits of priming for several combinations of parameters (costs of priming f_{prim}; columns, competition coefficient (α, 1 and 1.25) and feeding rate (*F*; 0-2.2e-07). The proportion of LAI that is consumed is constant during herbivory. The benefits of priming decreases when the season progresses because the relative costs of priming increase over the season.

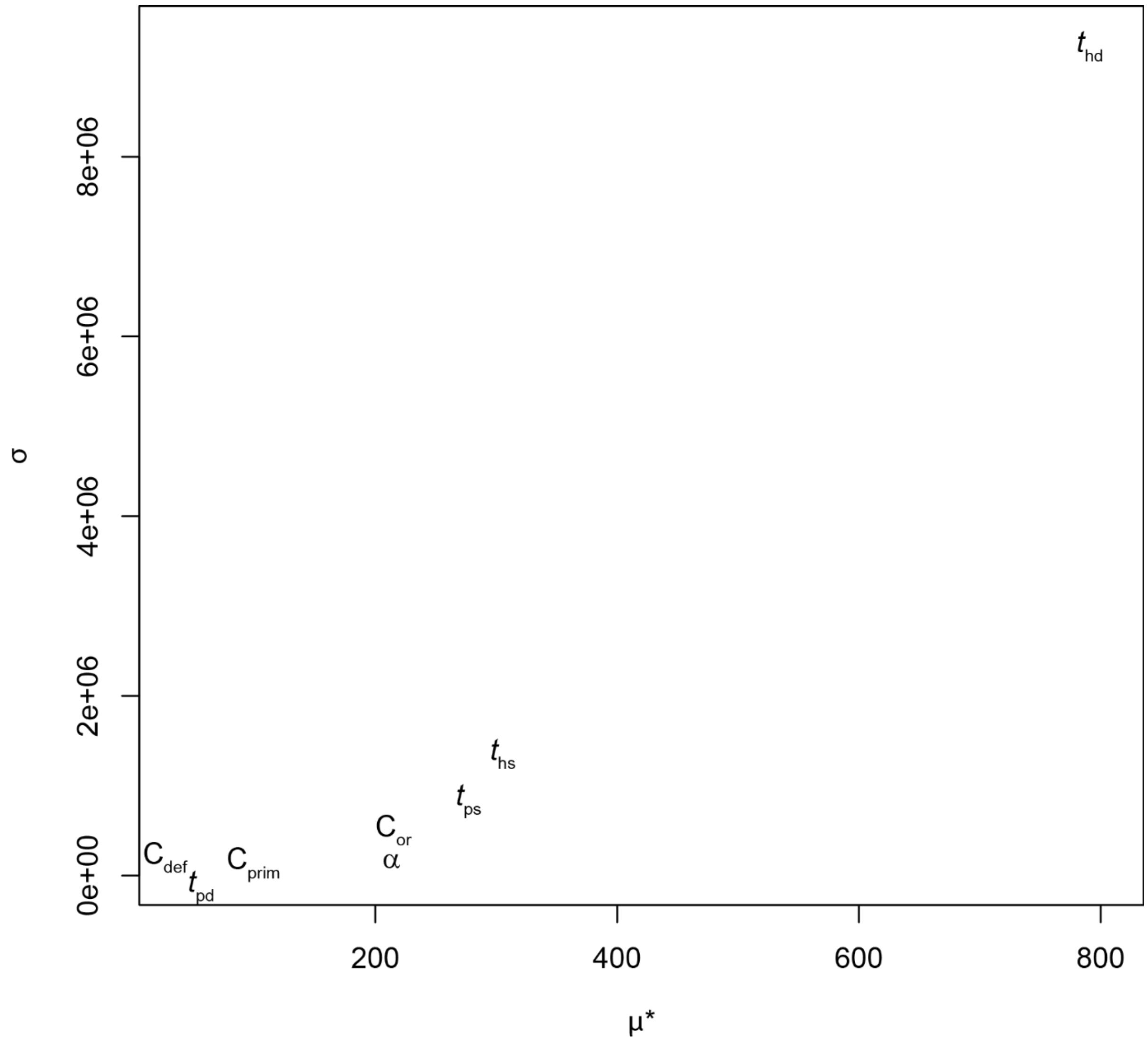

Fig. S2. Results of the Morris sensitivity analysis for eight parameters of the model. A high mean indicates a parameter that is an influential parameter for the output, a high variance indicates that this parameter is influential but that it depends on the value of other parameters, or because of a non-linear relationship of that parameter and the outcome variable. μ* is the mean of elementary effects for a given parameter. Elementary effects are calculated by the absolute change in the response variable over the change in the input value. σ is calculated as the variance of the elementary effects for a given parameter. For details on the Morris sensitivity method we refer to Wallach *et al.* (2014)

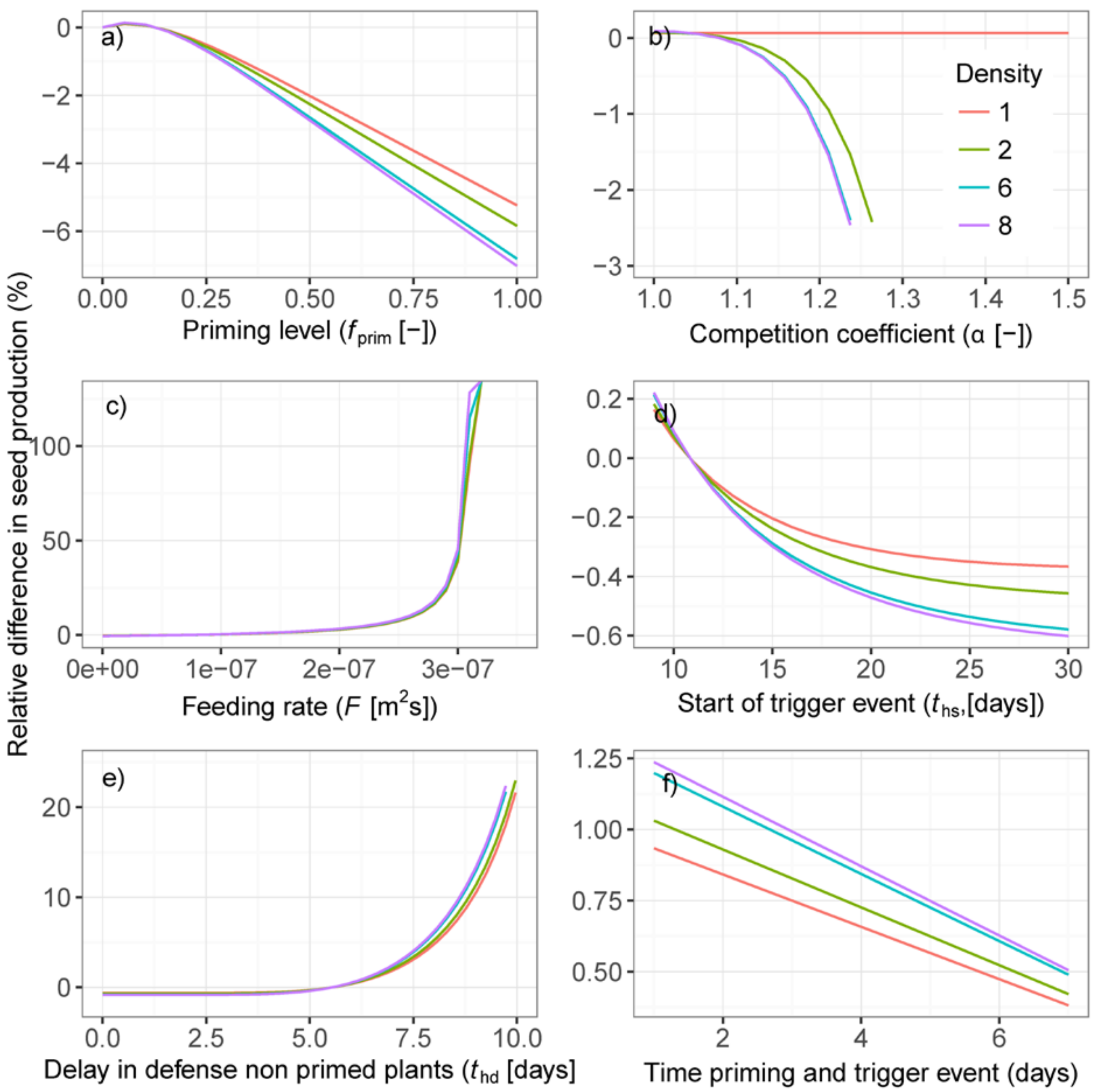

Figure S3. The relative change in seed production of a plant that primes for defense compared to naïve plants (used here as a proxy for the performance value of priming) as a function of a) the direct costs of priming ($\boldsymbol{f_{prim,i}}$), b) the competition coefficient (α), c) herbivore feeding rate, d) the day during the growing season herbivory starts, e) the delay in defense response of non-primed plants and f) the time between the trigger and the priming event. Priming is advantageous when the relative difference in seed production is larger than zero. A sigmoidal relationship between investment in defense and the reduction in feeding rate was assumed. Parameter settings: $t_{hs} = 10$, $t_{ps} = 2$, $t_{pd} = 8$, $\alpha = 1$, $C_{def} = 1$, $C_{prim} = 0.1$, $F_{pot} = 8e\text{-}08$, $t_{hd} = 6$, lognormal feeding pattern, priming response pattern =earlier (Fig. 2 main text), $t_f = 80$. Other parameter values are listed in Table 2.

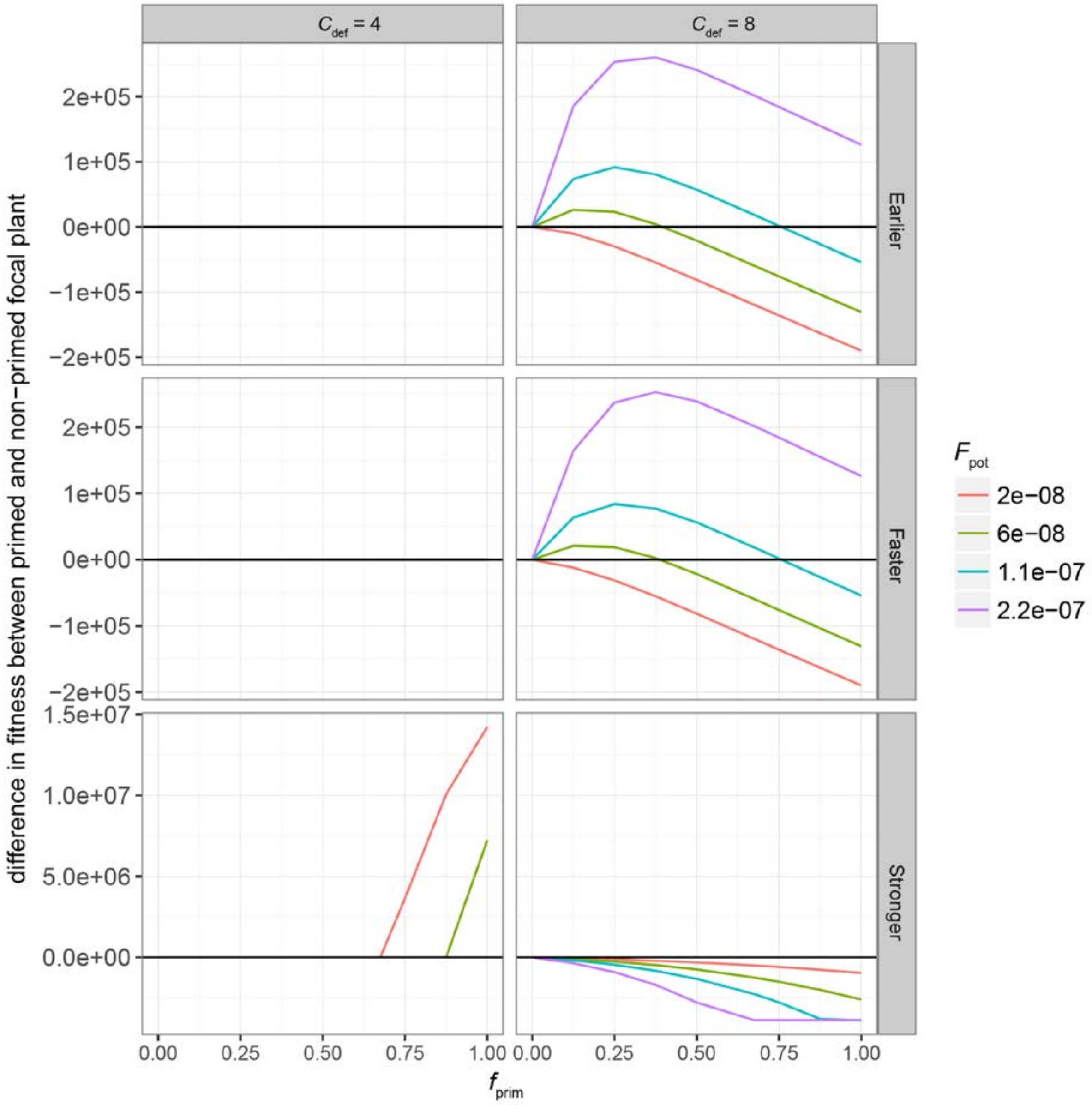

Figure S4. The difference in fitness of primed plants vs naïve plants for three response patterns (earlier, faster and stronger; rows) and two levels of defense: a suboptimal level of defense (C_{def} 4 µmol/m^{2}s) and an optimal level of defense (8 µmol/m^{2}s). When plants are suboptimally defended (left column) both the earlier and faster priming strategy and the non-priming strategy do not reach the reproductive phase. However, the priming response pattern "stronger" has higher defense levels compared to naïve plants and is able to reach the generative phase for the lowest two levels of feeding rate (F m^2 leaf s^{-1}; colours). When plants are optimally defended, it does not pay-off to invest more in defense compared to the optimal level. Hence, there is no benefit of the stronger response when defense levels are optimal (right lower corner).

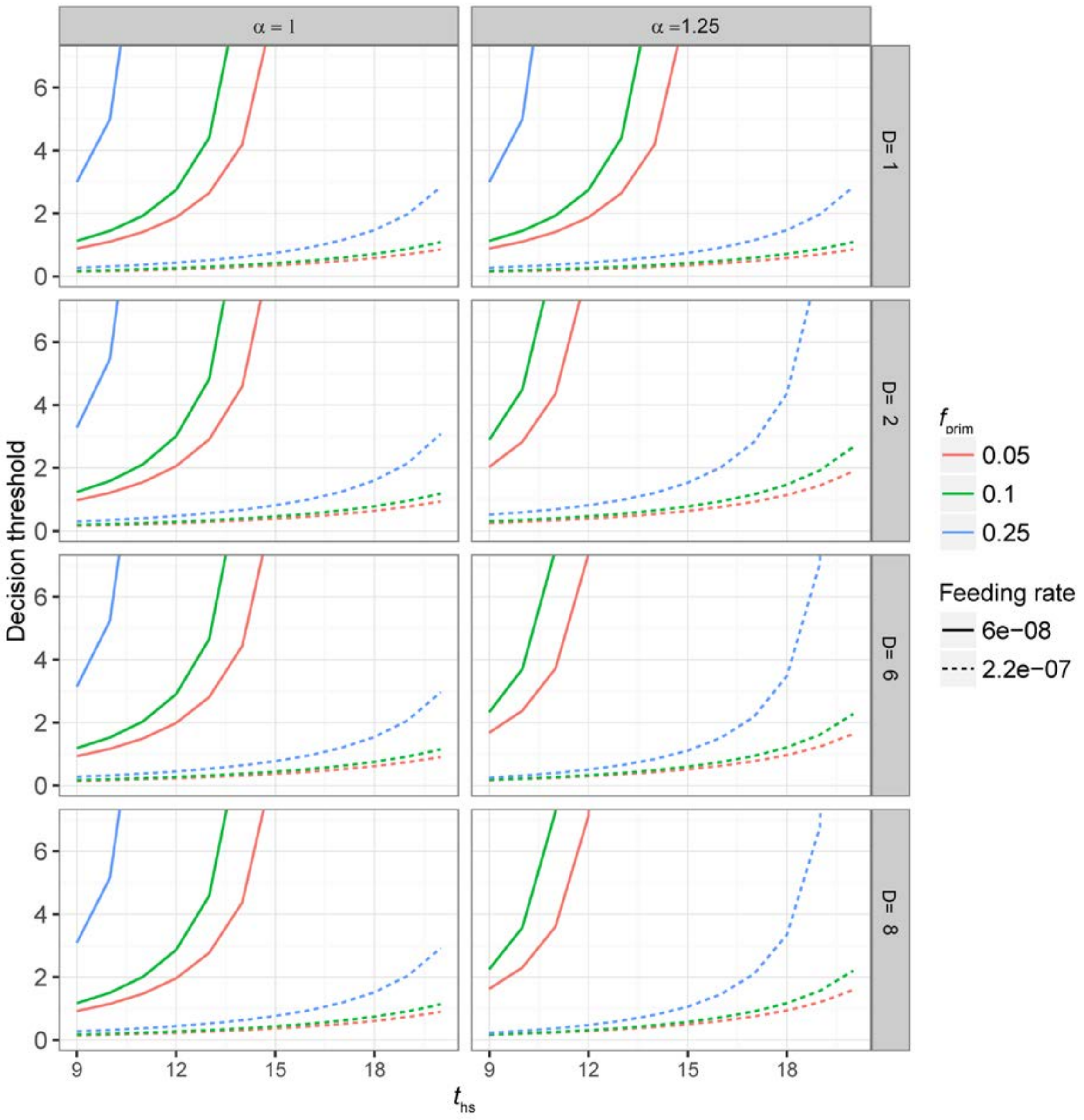

Figure S5. The relationship between the onset of herbivory and how much evidence is needed to prime for defense. When D is high, much evidence is needed that the cue signals future herbivore attack (for example through higher volatiles doses). We varied two feeding rates (6e-08 m^2/s and 2.2e-07 m^2/s), three levels of priming costs (0.05, 0.1 and 0.25), four plant densities (D=1,2,6,8), and the competition coefficient (α= 1, 1.25). The probability that the cue signals herbivory attack is 0.5. The decision threshold increases with t_{hs} which implies that plants should become less responsive to the a priming cue that signals a probability of attack of 0.5, because the costs of priming when the herbivore attack does not occur are larger compared to the costs of not priming when the herbivore does show up. When feeding rate is high, less evidence is needed that the priming cue signals future herbivore attack.

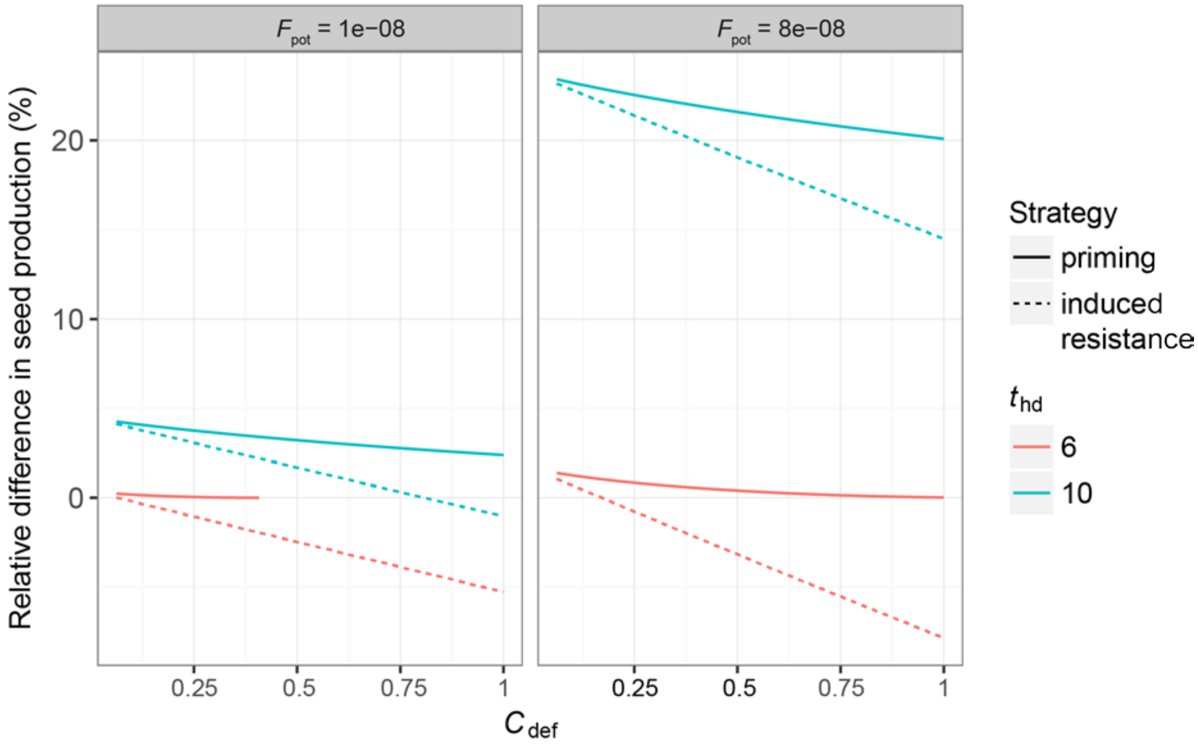

Figure S6. The relationship between the costs of defense and the benefit of the priming strategy (solid lines) relative to a non-priming strategy and the benefit of inducible resistance strategy in response to a priming cue relative to a non-priming strategy (dashed lines) for two values of t_{hd} (time needed to buildup defenses) and two feeding rates (panels). For the priming strategy, the level of priming (C_{prim}) was optimised for each level of C_{def}. For the inducible resistance strategy C_{prim} is one (see main text). Note that the red line in the left panel stops around C_{def} 3.5 because at higher values of C_{def} the optimal priming level is zero (which is equal to the non-priming strategy). Parameter settings: t_{hs} = 10, t_{ps} = 3, t_{pd} = 8, α = 1, C_{def} = 1, F_{pot} = 8e-07, t_{hd} = 6, lognormal feeding pattern, priming response pattern =earlier (Fig. 2), t_f = 80. Other parameter values are listed in Table 2.

References

Wallach D, Makowski D, Jones JW, Brun F. 2014. *Working with dynamic crop models - methods, tools, and examples for agriculture and environment*. London: Elsevier.
